# Regulatory T cells sabotage anti-tumor γδ T cells by creating IL-2–deficient environments

**DOI:** 10.1084/jem.20252133

**Published:** 2026-05-13

**Authors:** Rafael Blanco-Domínguez, André Miguel Vaz-Pinto, Leandro Barros, Noella Lopes, Beatriz Henriques-Alves, Mariana Carreira, Carlos Labão-Almeida, Julie C. Ribot, Gonçalo J.L. Bernardes, Bruno Silva-Santos, Sofia Mensurado

**Affiliations:** 1 Gulbenkian Institute for Molecular Medicine, Lisbon, Portugal; 2 Translational Chemical Biology Group, Spanish National Cancer Research Centre, Madrid, Spain; 3 Yusuf Hamied Department of Chemistry, University of Cambridge, Cambridge, UK; 4 Faculdade de Medicina da Universidade de Lisboa, Lisbon, Portugal

## Abstract

Regulatory T (Treg) cells are potent immunosuppressors of conventional αβ T cells in the tumor microenvironment, but how they may affect innate-like γδ T cells remains poorly understood. Here, we show that induced Treg depletion in mice selectively unleashes IFNγ-producing γδ T cells, which are required for tumor control in an orthotopic breast cancer model. Treg cells outcompete IFNγ^+^ γδ T cells for IL-2 due to increased expression of the high-affinity IL-2 receptor, thereby limiting the proliferation and effector functions of IFNγ^+^ γδ T cells. Consistently, in vivo neutralization of IL-2 alongside Treg depletion abrogates the induction of IFNγ^+^ γδ T cell responses, whereas administration of an IL-2Rβγ_c_ agonist circumvents Treg-mediated suppression and enhances tumor control. Finally, Treg cells also inhibit endogenous and expanded human γδ T-cells, which can be rescued by IL-2Rβγ_c_ agonism to enhance therapeutic responses in xenografted mice. Thus, bypassing Treg-mediated suppression may improve the outcome of γδ T cell–based immunotherapies.

## Teaser

Blanco-Domínguez et al. demonstrate that regulatory T cells constrain IFNγ-producing γδ T cells by competing for IL-2, thereby limiting their expansion and anti-tumor function. IL-2Rβγc agonism can overcome this suppression to enhance γδ T cell–mediated tumor control.

## Introduction

γδ T cells represent a conserved and unique lymphocyte lineage that bridges innate and adaptive immunity (Vantourout and Hayday, 2013). Unlike conventional αβ T cells, γδ T cells are not restricted by major histocompatibility complex (MHC)–mediated antigen presentation and often respond with innate-like dynamics, thus constituting nonredundant immune effectors attractive for immunotherapeutic exploitation (Hayday et al., 2024). We and others have shown that mouse γδ T cells are functionally heterogeneous, with two main effector subsets emerging in multiple models of infection or cancer: IFNγ-producing and IL-17–producing γδ T cells (Silva-Santos et al., 2019; Ribot et al., 2021). These distinct γδ T cell subsets are developmentally programmed in the thymus (Jensen et al., 2008; Ribot et al., 2009), home to many peripheral (lymphoid and nonlymphoid) tissues (Ribot et al., 2021), and employ different mechanisms of activation upon challenge (Inácio et al., 2025). In the context of cancer, IL-17^+^ γδ T cells have been associated with tumor progression, whereas IFNγ^+^ γδ cells inhibited tumor growth in various syngeneic mouse models (Gao et al., 2003; Silva-Santos et al., 2019; Lopes et al., 2021; Reis et al., 2022). Importantly, human γδ T cells are highly biased toward IFNγ (but not IL-17) production, which underscores their potential as effectors of cancer immunotherapy (Hayday et al., 2024; Silva-Santos et al., 2019).

Despite their well-established importance in cancer immunity, the functional regulation of γδ T cells, particularly IFNγ^+^ γδ T cells, within the tumor microenvironment (TME) remains poorly understood. Previous studies have mostly focused on cell-intrinsic inhibitory pathways, particularly immune checkpoints like PD-1, LAG-3, or TIM-3 (Hoeres et al., 2019; Gao et al., 2023). Concerning extrinsic factors, neutrophils have been shown to suppress IFNγ production by human γδ T cells in vitro (Sacchi et al., 2018; Sabbione et al., 2014), but the critical in vivo immune regulators of IFNγ^+^ γδ T cells remain to be identified, which may hold the key to enhancing their performance in cancer immunotherapy.

Regulatory T (Treg) cells, namely CD4^+^ T cells expressing the master transcription factor Foxp3, are well-established suppressors of effector αβ T cell responses in cancer (Tanaka and Sakaguchi, 2017). Removal of Treg cells enhances tumor control by increasing effector αβ T cell proliferation and cytokine production and is a key target of lymphodepletion strategies prior to adoptive cell transfer (Gattinoni et al., 2005; Wrzesinski et al., 2010). These strategies function in part by eliminating “cytokine sinks”—Treg cells and other IL-2/IL-15–consuming populations—to promote homeostatic expansion and function of the adoptively transferred effector T cells. Clinical observations further indicate that in the context of chimeric antigen receptor–transduced T (CAR-T) cell therapies, poor CAR-T cell expansion and persistence correlate with elevated intratumoral Treg levels (Good et al., 2022). Although different Treg-derived anti-inflammatory molecules have been shown to suppress γδ T cells in other contexts (Okeke and Uzonna, 2019), their influence on anti-tumor γδ T cell responses has never been decisively addressed in vivo.

In this study, we fill this gap by systematically evaluating the impact of Treg depletion on tumor-infiltrating γδ T cell populations using syngeneic cancer models. We demonstrate that Treg cells selectively suppress anti-tumor IFNγ-producing (but not pro-tumor IL-17–producing) γδ T cells by limiting IL-2 availability in the TME, a cytokine required for type 1 responses. This suppression can be reversed by the administration of an IL-2Rβγ_c_–selective agonist, which works not only in mice but also with human γδ T cells, notably enhancing the in vitro and in vivo functions of expanded γδ T cells used in adoptive cell therapy. Altogether, these findings uncover a novel immunoregulatory axis between Treg cells and anti-tumor γδ T cells, which can be targeted to enhance the activity of upcoming γδ T cell–based immunotherapies for cancer.

## Results

### Treg depletion unleashes IFNγ-producing γδ T cell responses

**Figure 1.**
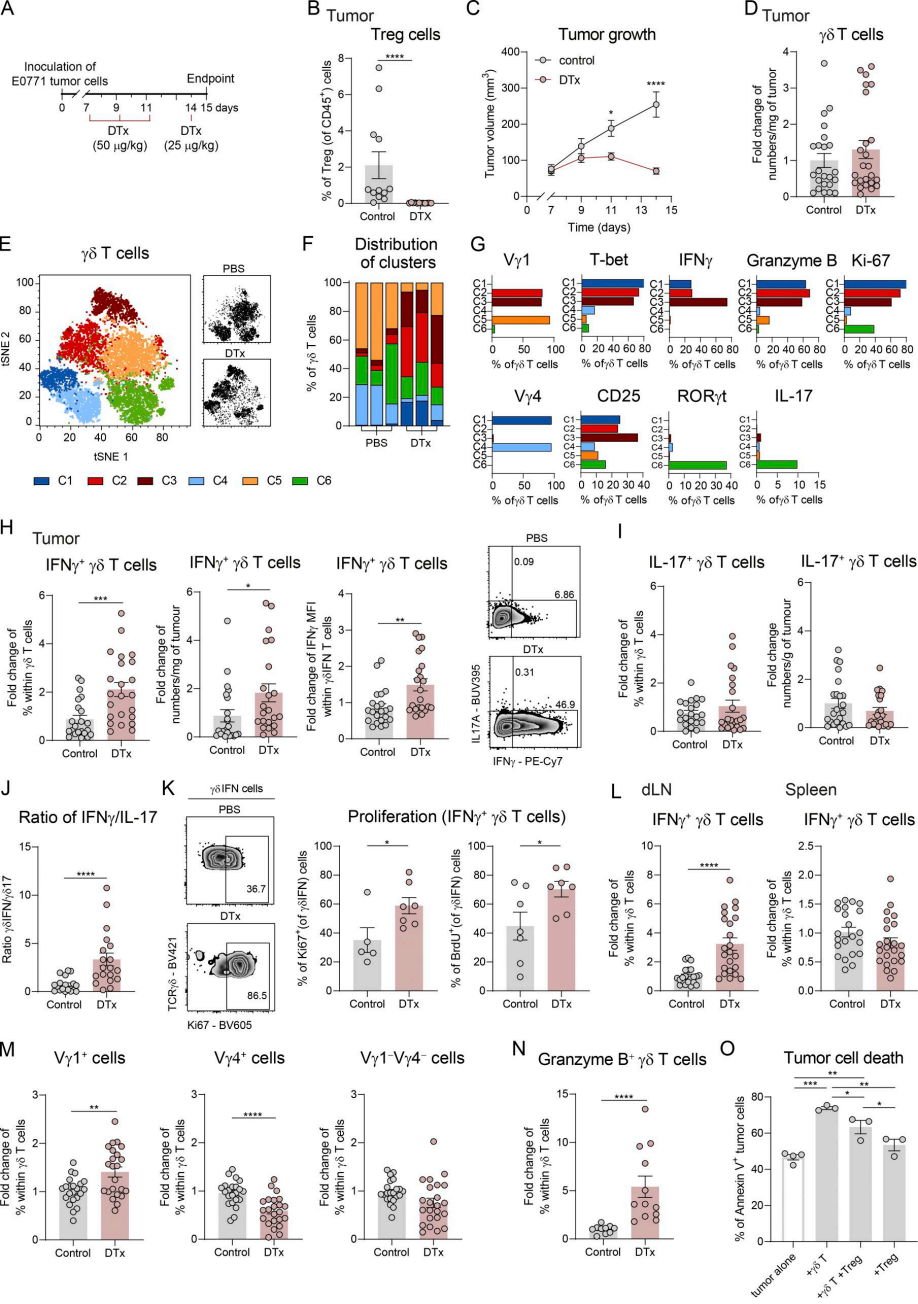
**Treg cell depletion unleashes IFNγ-producing γδ T cell responses. (A)** Schematic representation of the experimental approach. 1 × 10^6^ of E0771 breast cancer cells were inoculated subcutaneously in the mammary fat pad of Foxp3-DTR mice. DTx in PBS was administered intraperitoneally on days 7, 9, 11 (50 μg/kg), and 14 (25 μg/kg) after tumor inoculation. **(B)** Percentages of Treg cells in tumors at day 15 after tumor inoculation (*n* = 12 mice per group), represented as means ± SEM and analyzed by Mann–Whitney U test. **(C)** Tumor growth of PBS- or DTx-treated mice (*n* = 14 mice per group). Means ± SEM, repeated measures two-way ANOVA with Sidak’s multiple comparisons test. **(D)** Fold change of numbers of γδ T cells per milligram of tumor (*n* = 22 control and 23 DTx mice), normalized over controls, represented as means ± SEM and analyzed by the Mann–Whitney U test. Data in B–D represent a pool of three independent experiments. **(E)** Unsupervised hierarchical clustering of tumor-infiltrating γδ T cells from PBS (pool of *n* = 3 mice) or DTx-treated mice (pool of *n* = 3 mice), based on spectral flow cytometry data. **(F)** Distribution of clusters across the individual mice (*n* = 6). **(G)** Protein expression levels of different markers across clusters. Data in E–G represent one representative experiment out of >3 independent experiments. **(H)** Representative density plots (gated on γδ T cells) and quantification of IFNγ^+^ γδ T cells in tumors (*n* = 22 control and 23 DTx mice). Fold change of percentage, numbers per milligram, and mean fluorescence intensity (MFI) over controls are represented. **(I)** Fold change of percentage of IL-17A^+^ γδ T cells and numbers per milligram in tumors (*n* = 22 Control and 23 DTx mice). **(J)** γδ T cell polarization measured by the ratio of percentages of IFNγ^+^ versus IL-17A^+^ γδ T cells (γδIFN/γδ17) (*n* = 18 control and 19 DTx mice). Data in H–J are a pool of three different experiments. **(K)** Representative density plots (gated on IFNγ^+^ γδ T cells) and quantification of proliferation of tumor IFNγ^+^ γδ T cells, measured by Ki-67 (*n* = 5 control 7 DTx mice) and BrdU (*n* = 7 mice per group) staining. One representative out of two independent experiments. **(L)** Fold change of percentages of IFNγ^+^ γδ T cells in the tumor dLNs spleen of control and DTx-treated mice (*n* = 22 control and 23 DTx mice). **(M)** Fold change of percentages of Vγ subsets (within γδ T cells) in the tumors (*n* = 22 control and 23 DTx mice). Data in L and M are a pool of three different experiments. **(N)** Fold change of percentages of granzyme B^+^ cells within γδ T cells in tumors (*n* = 10 control and 12 DTx mice), one representative out of three independent experiments. Data in H-N represented as means ± SEM and analyzed by unpaired *t* test for normal distributions or Mann–Whitney U test for non-normal distributions. **(O)** Quantification of tumor cell death of E0771 cells over a 24-h killing assay in the presence of γδ T cells, Treg cells, or both, measured by percentage of annexin V ^+^ cells (*n* = 3–4 replicates). Data are represented as means ± SEM and analyzed by one-way ANOVA with Tukey’s post hoc test. One representative out of two independent experiments. *P < 0.05, **P < 0.01, ***P < 0.001, and****P < 0.001.

**Figure S1.**
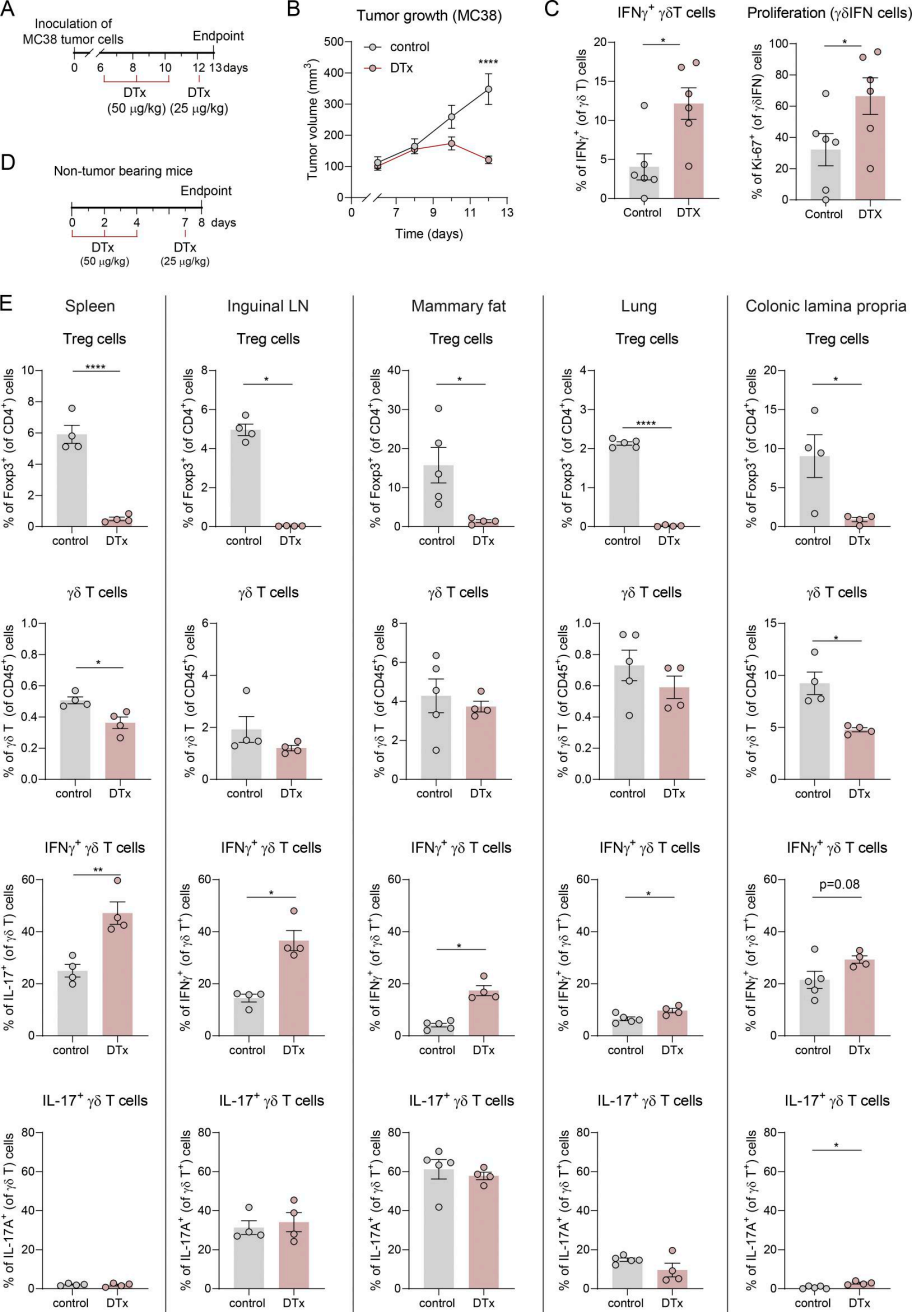
**IFNγ** ^***+***^ **γδ T cell expansion upon Treg cell depletion in MC38 colon cancer model and in tumor-free mice. (A)** Schematic representation of the experimental approach. 2 × 10^6^ of MC38 colon cancer cells were inoculated subcutaneously in the flank of Foxp3-DTR mice. DTx in PBS was administered intraperitoneally on days 6, 8, 10 (50 μg/kg), and 12 (25 μg/kg) after tumor inoculation. **(B)** Tumor growth of PBS- or DTx-treated mice (*n* = 6 mice per group). Means ± SEM, repeated measures two-way ANOVA with Sidak’s multiple comparisons test. **(C)** Quantification of the frequency of IFNγ^+^ γδ T cells and of proliferation of IFNγ^+^ γδ T cells, measured by Ki-67 (*n* = 6) staining, in tumors. **(D)** Schematic representation of the experimental approach. Foxp3-DTR mice were injected DTx in PBS on days 0, 2, 4 (1.5 μg), and 7 (0.75 μg). **(E)** Quantification of percentages of Treg cells and γδ T cells within CD45^+^ cells and quantification of IFNγ and IL-17A expression by γδ T cells in the spleen, inguinal LN, mammary fat, lung, and colonic lamina propria of DTx-treated or control mice. Data are represented as means ± SEM and analyzed by unpaired *t* test (for normal distributions) or Mann–Whitney U test (for non-normal distributions). Data are representative of two independent experiments. Data are represented as means ± SEM and analyzed by one-way ANOVA with Tukey’s multiple comparisons test. *P < 0.05, **P < 0.01, and ****P < 0.001.

To investigate the effects of Treg depletion on γδ T cell responses to tumors, we employed a syngeneic breast cancer model involving the orthotopic implantation of the E0771 breast cancer cell line into Foxp3-diphtheria toxin (DTx) receptor (DTR) mice, which express the human DTR under control of the *Foxp3* promoter. Sequential administration of DTx one week after tumor implantation, following confirmation of palpable tumors (Fig. 1 A), enabled transient and selective depletion of Treg cells across multiple tissues (Kim et al., 2007), including tumors (Fig. 1 B). In agreement with previous literature (Bos et al., 2013; Joshi et al., 2015; Simpson et al., 2013), Treg depletion led to tumor control (Fig. 1 C). While total numbers of γδ T cells remained unchanged (Fig. 1 D), Treg depletion induced a drastic phenotypic switch in tumor-infiltrating γδ T cell subsets (Fig. 1 E). Multicolor spectral flow cytometry analysis classified γδ T cells within tumors into six different clusters, revealing a pronounced expansion of those (C1–C3) presenting features of type 1 (IFNγ-producing) response in DTx-treated mice (Fig. 1, E–G). Consistently, the percentages and numbers of IFNγ^+^ γδ T cells, as well as the levels of IFNγ production by these cells (quantified as mean fluorescence intensity), were increased after Treg depletion (Fig. 1 H). By contrast, the levels of IL-17–producing γδ T cells remained virtually unchanged (Fig. 1, G and I), thus resulting in an increased IFNγ^+^/IL-17^+^ γδ T cell ratio (Fig. 1 J). Augmented Ki-67 expression and BrdU incorporation highlighted the increased proliferation of IFNγ^+^ γδ T cells underlying the expansion of this subset (Fig. 1 K). Importantly, we also observed a similar accumulation of proliferative IFNγ^+^ γδ T cells in a MC38 (colorectal) subcutaneous tumor model, correlative with an enhanced tumor control upon Treg cell depletion (Fig. S1, A–C), suggesting that this phenomenon is preserved across tumor models. The observed IFNγ^+^ γδ T cell expansion was more pronounced in tumors and tumor-draining LNs (dLNs) than in the spleen, suggesting a preferential effect in the tumor context (Fig. 1 L). By contrast, in tumor-free mice, IFNγ^+^ γδ responses were found systemically upon Treg depletion (Fig. S1 D and E). One possibility is that the TME locally retains or preferentially activates γδ T cells following Treg depletion, thereby restricting their systemic dissemination. In tumor-free mice, this constraint would be absent, allowing IFNγ^+^ γδ T cell responses to manifest systemically. However, the mechanisms underlying this phenomenon remain to be determined. Notably, the proportions of different TCRγ-specific subsets was altered in DTx-treated mice, with heightened representation of Vγ1^+^ cells in the tumors, compared with decreased Vγ4^+^ cells and unchanged Vγ1^−^Vγ4^−^ cells (Fig. 1 M). In addition to their upregulation of IFNγ expression in the absence of Treg cells, tumor-infiltrating γδ T cells also increased their expression of the cytotoxic enzyme, granzyme B (Fig. 1 N), supporting enhanced anti-tumor potential. To demonstrate a direct impact of Treg cells on γδ T cell cytotoxicity, we co-cultured them for 24 h with E0771 breast cancer cells and observed impaired tumor-cell killing by γδ T cells when in the presence of Treg cells (Fig. 1 O). These data demonstrate that Treg cells selectively suppress type 1 cytotoxic, but not type 17, γδ T cell responses to tumors.

### IFNγ-biased Vγ1^+^ γδ T cells are anti-tumor effectors of Treg depletion therapy

**Figure S2.**
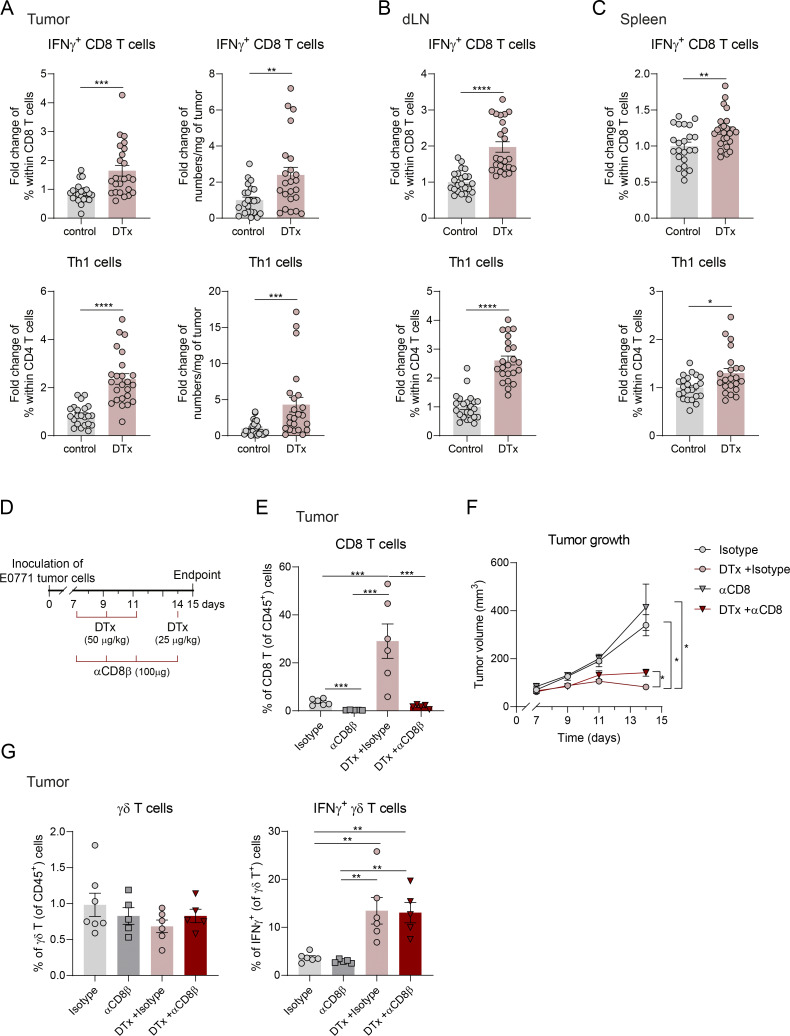
**Treg cell depletion increases systemic IFNγ-producing αβ T cells and reveals the anti-tumor** **contribution** **of CD8 T cells. (A–C)** Quantification of IFNγ^+^ CD8 and CD4 (Th1) T cells as a fold change of percentage and numbers per milligram in (A) tumors or as a fold change of percentages in (B) dLNs and (C) spleen of DTx-treated over control mice. Data are represented as means ± SEM of a pool of three independent experiments and analyzed by unpaired *t* test for normal distributions or Mann–Whitney U test for non-normal distributions. **(D)** Schematic representation of the experimental approach. 1 × 10^6^ of E0771 breast cancer cells were inoculated in the mammary fat pad of Foxp3-DTR mice. DTx in PBS was administered intraperitoneally on days 7, 9, 11 (1.5 μg), and 14 (0.75 μg) after tumor inoculation. On the same days, 100 μg of αCD8β mAb or isotype control was administered intraperitoneally. **(E)** Percentages of CD8 T cells (within CD45^+^ T cells) in tumors (*n* = 6 isotype, 5 DTx + isotype, 6 αCD8β, and 5 DTx + αCD8β). Data are represented as means ± SEM and analyzed by one-way ANOVA with Tukey’s multiple comparisons test. **(F)** Tumor growth of mice treated with isotype (*n* = 7), αCD8β (*n* = 5), DTx + isotype (*n* = 6), or DTx + αCD8β (*n* = 5), analyzed by repeated measures two-way ANOVA with Sidak’s multiple comparisons test. **(G)** Percentages of γδ T cells (within CD45^+^ T cells) and IFNγ+ within γδ T cells (*n* = 6 isotype, 5 DTx + isotype, 6 αCD8β, and 5 or DTx + αCD8β) in tumors. Data are representative of two independent experiments, are represented as means ± SEM, and analyzed by one-way ANOVA with Tukey’s multiple comparisons test. *P < 0.05, **P < 0.01, ***P < 0.001, and ****P < 0.001.

**Figure 2.**
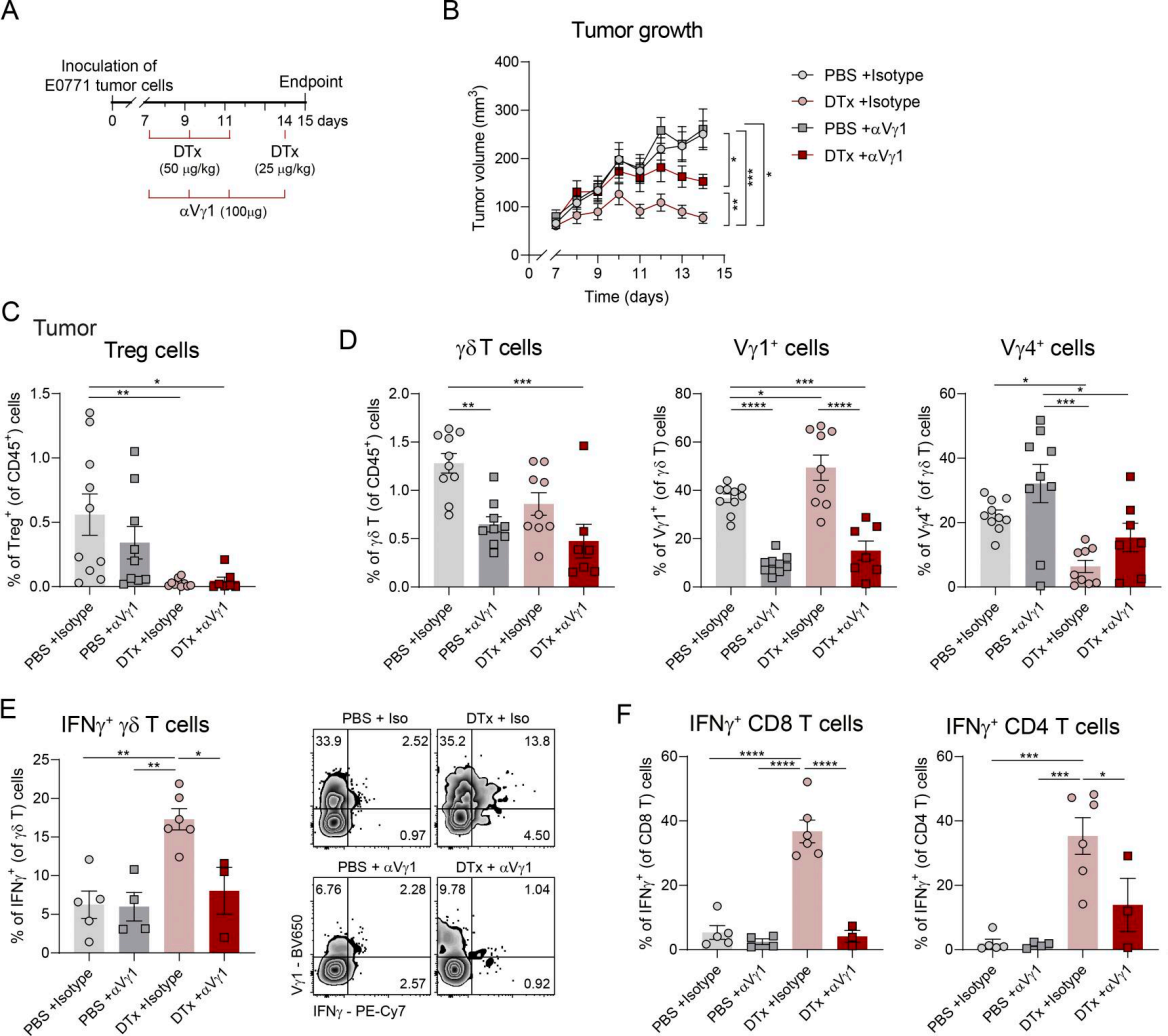
**IFNγ-biased Vγ1** ^**+**^ **γδ T cells are anti-tumor effectors of Treg depletion therapy.** **(** **A** **)** Schematic representation of the experimental approach. 1 × 10^6^ of E0771 breast cancer cells were inoculated in the mammary fat pad of Foxp3-DTR mice. DTx in PBS was administered intraperitoneally on days 7, 9, 11 (1.5 μg), and 14 (0.75 μg) after tumor inoculation. On the same days, 100 μg of αVγ1 mAb or isotype control was administered intraperitoneally. **(B)** Tumor growth of mice treated with PBS + isotype (*n* = 10), DTx + isotype (*n* = 9), PBS + αVγ1 (*n* = 9), or DTx + αVγ1 (*n* = 7). Pool of two independent experiments, repeated measures two-way ANOVA with Sidak’s multiple comparisons test. **(C)** Percentages of Treg cells in tumors at day 15 after tumor inoculation. **(D)** Percentages of γδ T cells (within CD45^+^ T cells) and Vγ1^+^ and Vγ4^+^ cells within γδ T cells (*n* = 10 PBS + isotype, 9 DTx + isotype, 9 PBS + αVγ1, and 7 DTx + αVγ1). Data in C and D, represented as means ± SEM, are a pool of two independent experiments and analyzed by one-way ANOVA with Tukey’s multiple comparisons test. **(E)** Percentage of IFNγ^+^ cells within γδ T cells and representative density plots. **(F)** Percentage of IFNγ-producing CD4 and CD8 αβ T cells. Data in E and F, represented as means ± SEM (*n* = 5 PBS + isotype, 4 DTx + isotype, 6 PBS + αVγ1, and 3 DTx + αVγ1), are representative of two independent experiments and were analyzed by one-way ANOVA with Tukey’s multiple comparisons test. *P < 0.05, **P < 0.01, ***P < 0.001, ****P < 0.001.

Since Treg cell depletion also induces type 1 responses in αβ T cells (Fig. S2, A–C), we next aimed to evaluate the specific contribution of IFNγ^+^ γδ T cells to the observed tumor control. As Vγ1^+^ cells were the main IFNγ-producing γδ T cell subset expanded upon Treg elimination (Fig. 1 M), we administered (intraperitoneally) an anti-Vγ1 TCR monoclonal antibody (αVγ1 mAb) together with the DTx treatment to selectively deplete Vγ1^+^ γδ T cells (Zheng et al., 2017) (Fig. 2 A). Notably, depletion of Vγ1^+^ cells partially reversed the tumor control achieved by Treg depletion, resulting in increased tumor growth, which demonstrated a nonredundant role for Vγ1^+^ cells in the anti-tumor response elicited by Treg ablation (Fig. 2 B). As expected, αVγ1 mAb treatment did not significantly affect the frequency or extent of Treg depletion (Fig. 2 C) but effectively reduced the proportion of intratumoral Vγ1^+^ cells by approximately two-thirds (Fig. 2 D). Importantly, this intervention reduced the frequency of IFNγ^+^ γδ T cells in DTx-treated mice to levels observed in control animals (Fig. 2 E), thus supporting Vγ1^+^ cells as the critical IFNγ^+^ γδ T cells in this context. Most interestingly, transient Vγ1^+^ cell depletion also resulted in reduced frequencies of IFNγ-producing CD4^+^ and CD8^+^ αβ T cells, supporting a model in which Vγ1^+^ γδ T cells orchestrate IFNγ-based anti-tumor immunity unleashed following Treg depletion (Fig. 2 F), consistent with the established γδ T cell ability to modulate broader immune responses (Arias-Badia et al., 2024). While CD8^+^ T cells also contribute to tumor control following Treg depletion (Fig. S2, D–F), CD8 depletion did not affect γδ T cell responses (Fig. S2 G), indicating that IFNγ^+^ γδ T cells act nonredundantly and upstream in this anti-tumor immune cascade. Whether this effect is mediated directly by γδ T cell–derived IFNγ remains to be determined, as formally addressing this question would require conditional ablation of IFNγ specifically in γδ T cells. These data position IFNγ-producing Vγ1^+^ γδ T cells as effectors of anti-tumor immunity upon Treg depletion therapy.

### Treg depletion enhances IL-2 signaling on tumor-infiltrating IFNγ^+^ γδ T cells

**Figure S3.**
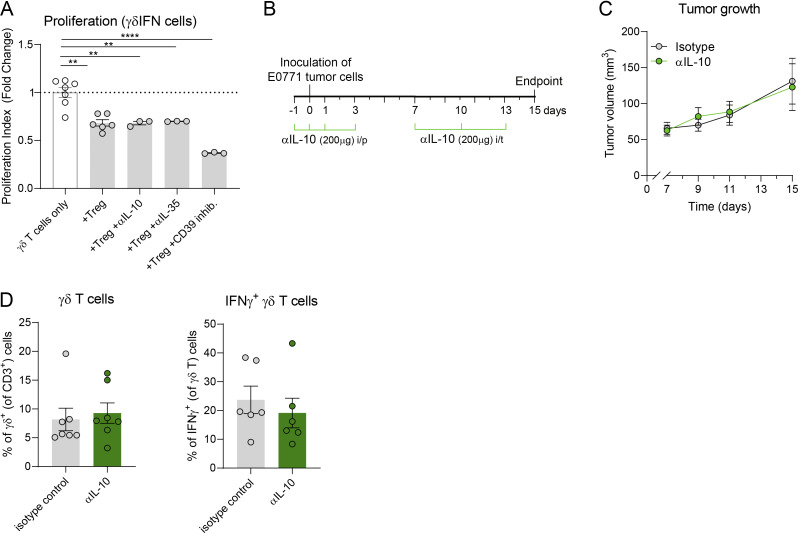
**Inhibition of IL-10, IL-35, and adenosine pathways does not impact Treg-mediated suppression of IFNγ** ^**+**^ **γδ T cells. (A)** Quantification of proliferation index of IFNγ^+^ γδ T cells in the presence or absence of Treg cells and in the presence of αIL-10– or αIL-35–neutralizing antibody or the CD39 inhibitor ARL67156 (fold change over γδ T cells only is represented). Data are representative of two independent experiments. **(B)** Schematic representation of the experimental approach. 1 × 106 of E0771 breast cancer cells were inoculated in the mammary fat pad of Foxp3-DTR mice (without any DTx administration). In addition, 200 μg of αIL-10–neutralizing antibody was administered intraperitoneally on days −1, 0, 1, and 3 relative to tumor inoculation, followed by intratumoral injections on days 7, 10, and 13. **(C)** Tumor growth of αIL-10–treated or control mice (*n* = 7 mice per group). Data are analyzed by repeated measures two-way ANOVA with Sidak’s multiple comparisons test without reaching significant differences between the two groups. **(D)** Percentages of γδ T cells within CD3^+^ T cells (*n* = 7 mice per group) and IFNγ^+^ cells within γδ T cells (*n* = 6 mice per group). Data are represented as means ± SEM and analyzed by unpaired *t* test. **P < 0.01 and ****P < 0.001.

**Figure S4.**
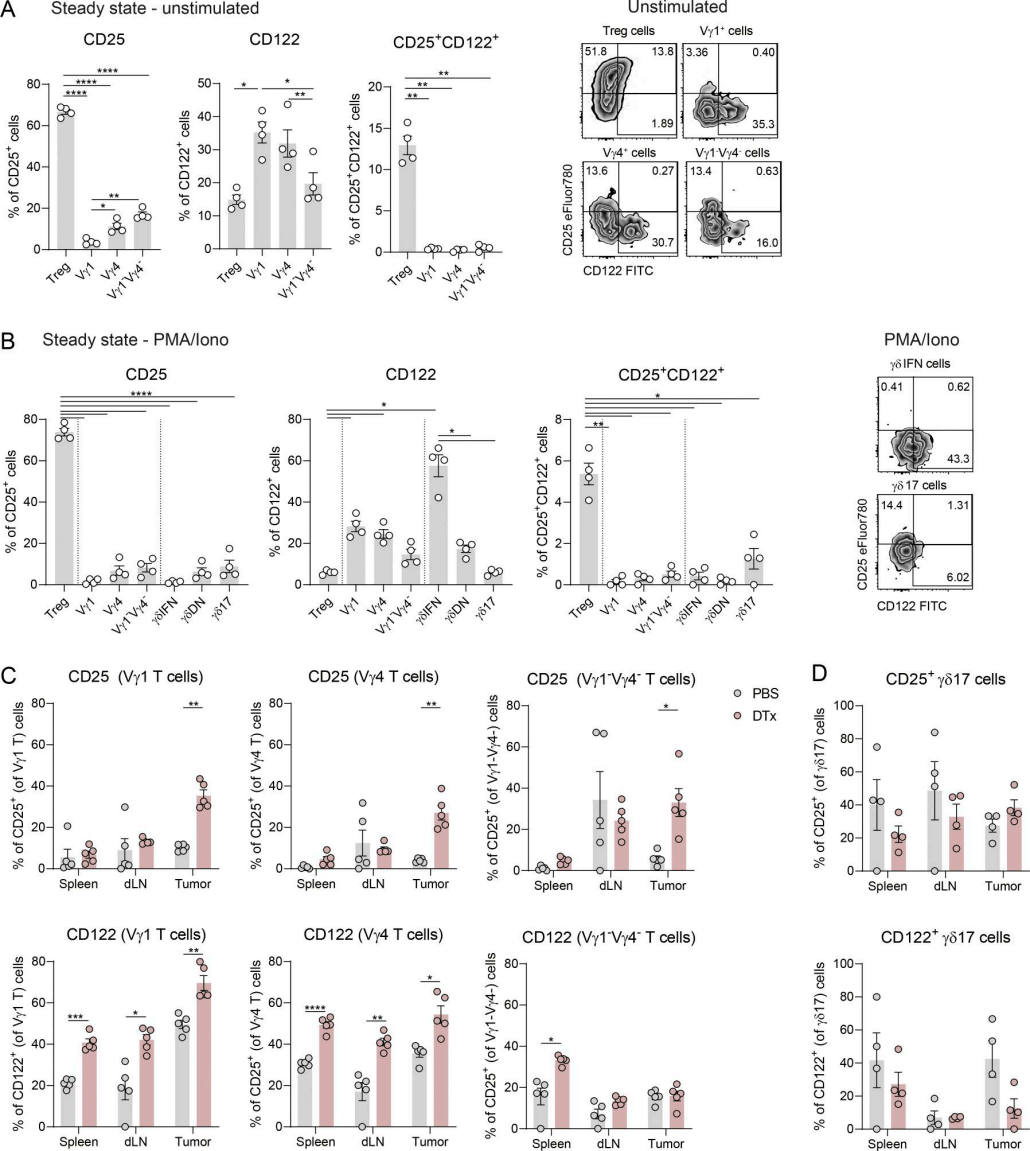
**Differential IL-2R expression in Treg cells and γδ T cell subsets. (A)** CD25 and CD122 expression in Treg cells and Vγ1^+^, Vγ4^+^, and Vγ1^−^Vγ4^−^ cells in steady state in peripheral LNs. **(B)** Comparative CD25 and CD122 expression in Treg cells, Vγ1^+^, Vγ4^+^, and Vγ1^−^Vγ4^−^ cells, as well as in Treg cells, IFNγ^+^ (γδIFN), IL-17A^+^ (γδ17), and IFNγ^−^IL-17A^−^ (γδDN) cells in LNs of steady-state mice after 3 h of stimulation with PMA/ionomycin. Data in A and B are represented as means ± SEM (*n* = 4 mice per group), analyzed by one-way ANOVA with Tukey’s multiple comparisons test and represent one out of two independent experiments. **(C)** CD25 and CD122 expression in Vγ1^+^, Vγ4^+^, and Vγ1^−^Vγ4^−^ cells from different organs of tumor-bearing mice after PMA/ionomycin. **(D)** CD25 and CD122 expression in γδ17 cells from different organs of tumor-bearing mice after PMA/ionomycin. Data in C and D are represented as means ± SEM (*n* = 4–5 mice per group), analyzed by two-way ANOVA with Sidak’s multiple comparisons test and represent one out of >3 independent experiments. *P < 0.05, **P < 0.01, ***P < 0.001, and ****P < 0.001.

To investigate how Treg cells suppress anti-tumor γδ T cells, we co-cultured sorted γδ T cells with Treg cells in the presence of αCD3/αCD28 stimulation under defined conditions targeting key immunosuppressive pathways employed by Treg cells (Tanaka and Sakaguchi, 2017). We found the presence of Treg cells directly inhibited the proliferation of IFNγ^+^ γδ T cells. However, neither blockade of Treg-derived IL-10 or IL-35 nor inhibition of adenosine production restored IFNγ^+^ γδ T cells proliferation (Fig. S3 A). In line with this, in vivo IL-10 neutralization neither enhanced IFNγ^+^ γδ T cell responses nor improved tumor control (Fig. S3, B–D). We cannot rule out a contribution of IL-10 to Treg-mediated suppression in other contexts, as the effect of this cytokine is highly variable across tissue environments. In the experimental system employed, our findings suggest that Treg-mediated suppression of IFNγ^+^ γδ T cells is independent from IL-10, IL-35, or adenosine. On the other hand, the increased expression of IL-2 receptor α (IL-2Rα/CD25) on IFNγ^+^ γδ T cell clusters in tumors lacking Treg cells (Fig. 1 G) could reflect enhanced IL-2 signaling. This observation is particularly relevant because a well-established mechanism of Treg suppression of both αβ T cells and NK cells is IL-2 deprivation (Chinen et al., 2016). Treg cells constitutively express the high-affinity trimeric IL-2 receptor, composed of IL-2Rα (CD25), IL-2Rβ/IL-15Rβ (CD122), and the common γ chain (IL-2Rγ/CD132). In contrast, effector αβ T cells lack CD25 expression at steady state, resulting in lower receptor affinity for IL-2. Consequently, Treg cells effectively outcompete effector cells for limited IL-2 availability, thereby constraining their activation and function (Kastenmuller et al., 2011). In agreement with previous reports (Corpuz et al., 2017; Shibata et al., 2008), we found that peripheral IFNγ-committed γδ T cells at steady-state express higher levels of CD122 compared with IL-17–committed subsets, which in turn show a relative preference for CD25 expression, although not to the level observed in Treg cells. Co-expression of CD25 and CD122 remained largely restricted to Treg cells (Fig. S4 A). Importantly, IL-2 receptor expression was not significantly altered following 3-h PMA/ionomycin stimulation, allowing reliable assessment of cytokine-producing effector subsets (Fig. S4 B).

**Figure 3.**
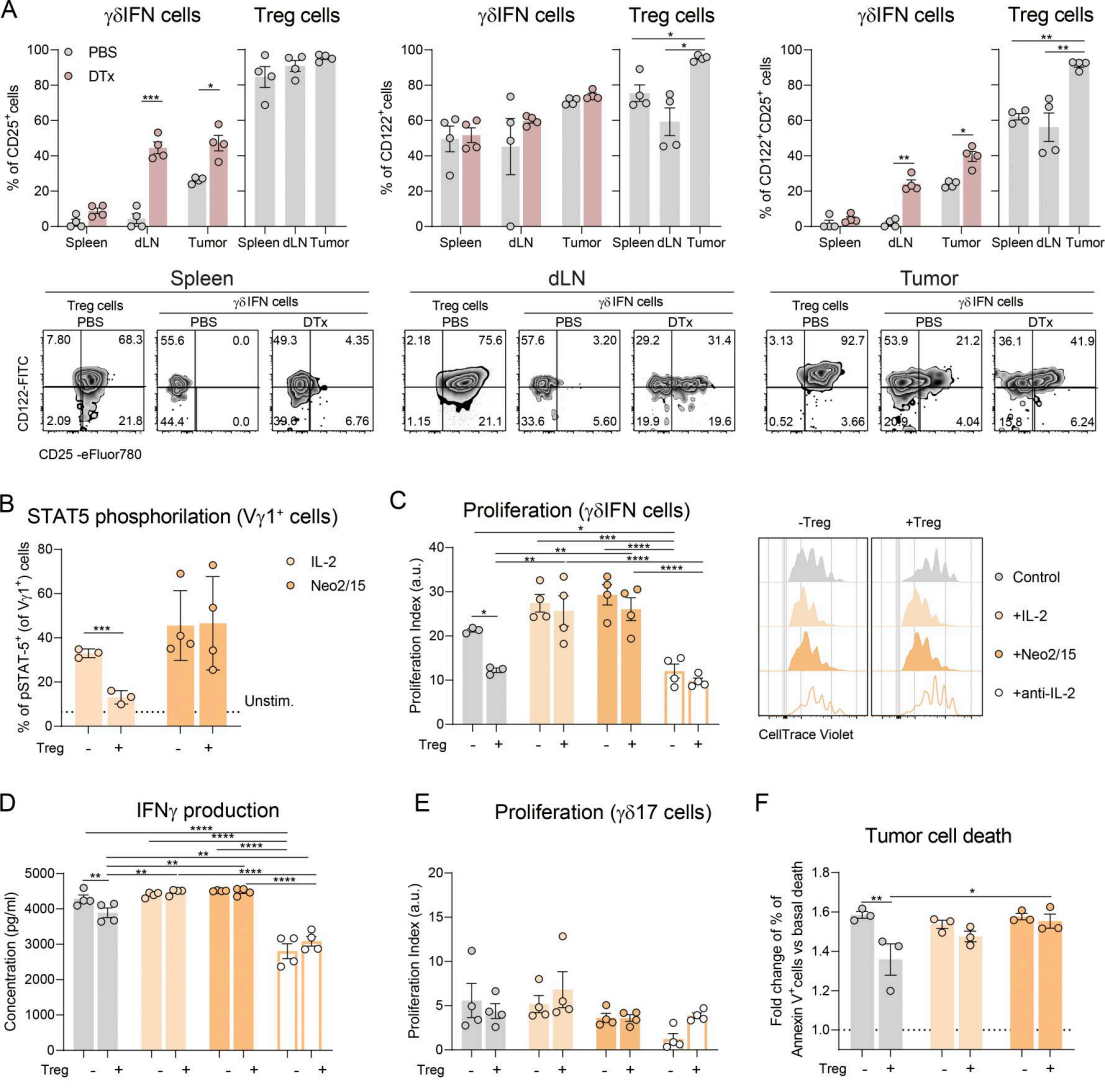
**Treg cells suppress IFNγ-producing γδ T cells by draining IL-2**. **(A)** Quantification and representative density plots of CD25 and CD122 expression by IFNγ^+^ γδ T cells and Treg cells in spleen, dLN, and tumors of DTx- (only IFNγ^+^ γδ T cells) and PBS-treated tumor-bearing mice (*n* = 4 mice per group). For IFNγ^+^ γδ T cells, data were analyzed by two-way ANOVA with Sidak’s multiple comparisons test; for Treg cells, by one-way ANOVA with Tukey’s multiple comparisons test. Data represent one experiment out of three independent experiments. **(B)** Quantification of STAT5 phosphorylation, measured by flow cytometry, in Vγ1^+^ γδ T cells after a 10-min pulse with IL-2 or Neo2/15 in the presence or absence of Treg cells (*n* = 3 replicates). The dotted line depicts the percentage of pSTAT5^+^ cells in the absence of stimulus. One representative out of three independent experiments. **(C)** Representative histograms of CellTrace Violet dilution and quantification of proliferation index of IFNγ^+^ γδ T cells, stimulated with plate bound anti-CD3 and soluble anti-CD28 mAbs, in the presence or absence of Treg cells and/or supplementation with IL-2, Neo2/15, or αIL-2 neutralizing antibody. **(D)** Concentration of IFNγ in the supernatants of the above-mentioned cultures, measured by ELISA. **(E)** Proliferation index of IL-17A+ γδ T cells in the presence or absence of Treg cells and/or supplementation with IL-2, Neo2/15, or αIL-2–neutralizing antibody. **(F)** Quantification of E0771 cell death over a 24-h-killing assay in the presence of γδ T cells, Treg cells. or both, measured by fold change of percentage of annexin V^+^ tumor cells over basal tumor death. Data in C–F represent means ± SEM (*n* = 3–4 replicates), are representative of >3 independent experiments, and were analyzed by two-way ANOVA with Sidak’s multiple comparisons test. All quantifications show means ± SEM. *P < 0.05, **P < 0.01, ***P < 0.001, and ****P < 0.001.

After receiving activation signals 1 and 2, autocrine IL-2 induces STAT-5 phosphorylation and subsequent CD25 expression in effector T cells to gain affinity for IL-2 and boost expansion and effector function (Shouse et al., 2024). This mechanism is particularly important for IFNγ^+^ γδ T cells, whose effector function is promoted by IL-2 signaling, and less so for IL-17–producing subsets (Ribot et al., 2012). In tumor-bearing mice, IFNγ^+^ γδ T cells exhibited higher levels of CD25 within tumors than in the periphery; however, they were still far from those found on Treg cells. Strikingly, Treg depletion upregulated CD25 expression on IFNγ^+^ γδ T cells in the tumors and tumor dLNs (but not in the spleen) (Fig. 3 A), where an increased frequency of IFNγ^+^ γδ T cells was also observed (Fig. 1, H and L), suggesting that enhanced IL-2 signaling accompanies the local expansion of these effector cells in the absence of Treg cells. IL-2R upregulation was observed across Vγ-based γδ T cell subtypes in DTx-treated mice (Fig. S4 C). By contrast, the levels of the IL-2R on IL-17^+^ γδ T cells remained unchanged across the different tissues, including tumors, following Treg depletion (Fig. S4 D). These data suggest that the presence of Treg cells may constrain IL-2 signaling in IFNγ^+^ (but not IL-17^+^) γδ T cells in tumors and tumor dLNs.

### Treg cells suppress IFNγ^+^ γδ T cells via IL-2 deprivation in vitro

**Figure S5.**
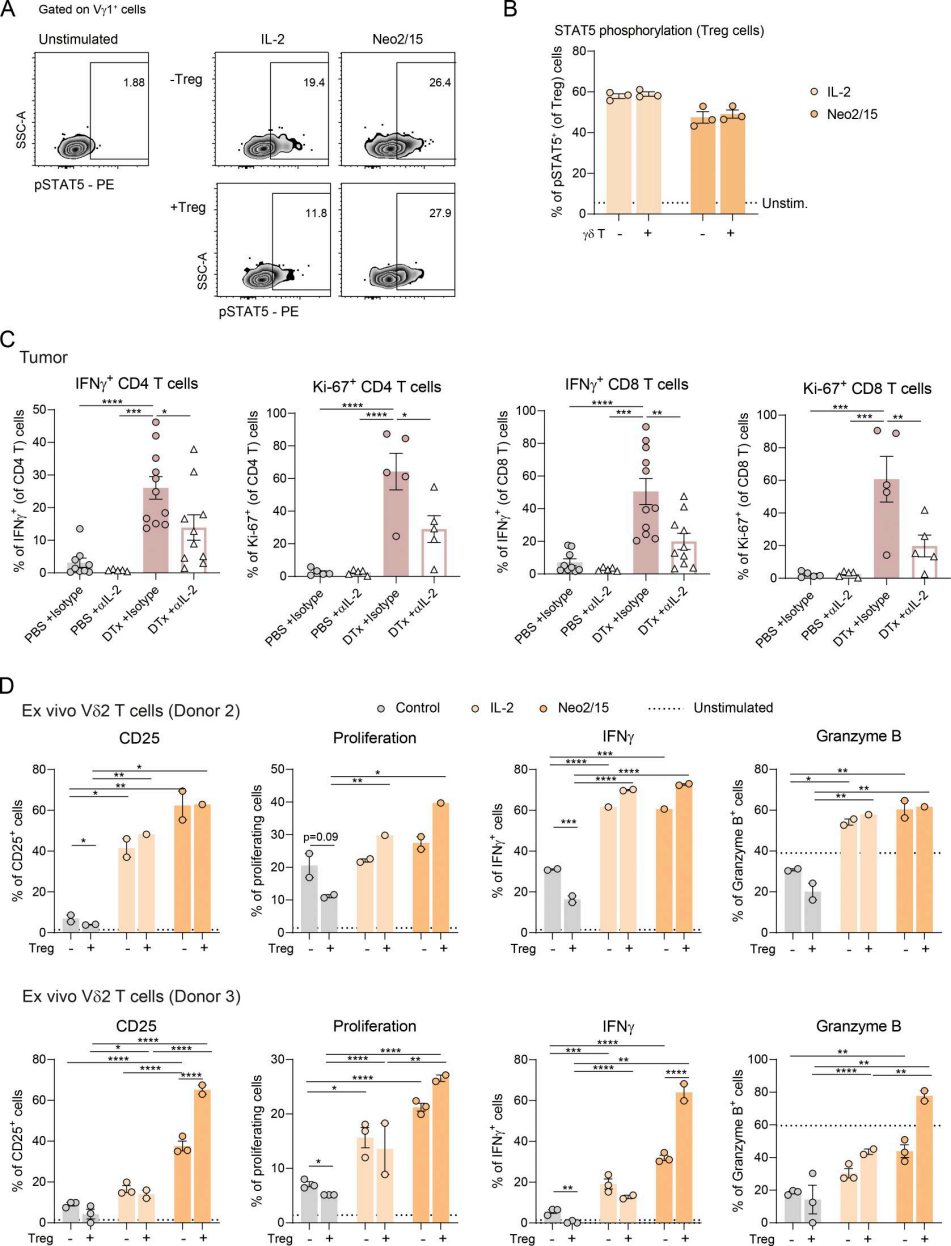
**IL-2-dependent suppression of γδ T and αβ T cells. (A and B)** IL-2 signaling in γδ and Treg cells. **(A)** Representative density plots of phosphorylated STAT5 expression on Vγ1^+^ T cells with or without IL-2 or Neo2/15. **(B)** Quantification of STAT5 phosphorylation, measured by flow cytometry, in Treg cells after a 10-min pulse with IL-2 or Neo2/15 in the presence or absence of γδ T cells (*n* = 3–4 replicates). The dotted line depicts the percentage of pSTAT5^+^ cells in the absence of stimulus. Means ± SEM of one representative out of three independent experiments. Data were analyzed by two-way ANOVA with Sidak’s multiple comparisons test although no significant differences were observed. **(C)** Effect of IL-2 neutralization on αβ T cell responses. Quantification of IFNγ and Ki-67 expression by tumor-infiltrating CD8 and CD4 T cells from mice treated with PBS + isotype-, DTx + isotype-, PBS + αIL-2–, or DTx + αIL-2–treated mice, measured by flow cytometry (IFNγ, *n* = 5–11 mice; Ki-67, *n* = 5). Data are represented as means ± SEM, are a pool of two independent experiments, and analyzed by one-way ANOVA with Tukey’s multiple comparisons test. **(D)** Inhibition of human γδ T cells by Treg cells across multiple donors. Quantification of proliferating sorted Vδ2^+^ T cells (measured by dilution of CellTrace Violet staining), as well as their CD25, IFNγ, and granzyme B expression after 3 days in culture with plate bound anti-CD3 and soluble anti-CD28 mAbs, with or without sorted Treg cells from autologous human PBMCs and in the presence or absence of IL-2 or Neo2/15. Dotted line represents baseline levels of unstimulated cells (no anti-CD3/28). Each donor corresponds to an independent experiment repeated a total of three times. *P < 0.05, **P < 0.01, ***P < 0.001, and ****P < 0.001.

To directly assess Treg interference with IL-2 signaling on γδ T cells, we co-cultured sorted Treg cells and γδ T cells and exposed them to a 10-min pulse of either recombinant IL-2 or the IL-2Rβγ_c_–selective agonist neoleukin-2/15 (Neo2/15), which activates IL-2R signaling independently of CD25 (Silva et al., 2019). In the absence of Treg cells, both IL-2 and Neo2/15 elicited robust and comparable STAT5 phosphorylation in IFNγ-committed (Vγ1^+^) γδ T cells, demonstrating that Neo2/15 can act directly on this subset. Notably, the presence of Treg cells selectively suppressed IL-2–driven STAT5 activation, but allowed for Neo2/15-induced signaling in Vγ1^+^ γδ T cells (Fig. 3 B and Fig. S5 A). Of note, STAT5 phosphorylation levels in Treg cells remained unchanged regardless of the presence of γδ T cells (Fig. S5 B). These findings support the notion that IL-2 preferentially binds to the high-affinity trimeric IL-2 receptor on Treg cells, whereas Neo2/15 signaling on γδ T cells circumvents this advantage.

In extended 3-day co-cultures with αCD3/αCD28 stimulation (that induces IL-2 production by IFNγ^+^ γδ T cells [Ribot et al., 2012]), we confirmed that IFNγ^+^ γδ T cells are directly suppressed by Treg cells (Fig. 3, C and D). Importantly, supplementation with either exogenous IL-2 or Neo2/15 reversed Treg-mediated suppression of both proliferation and IFNγ production by IFNγ^+^ γδ T cells (Fig. 3, C and D). Neutralization of endogenous IL-2 recapitulated the suppressive effects of Treg cells, and the addition of Treg cells did not further inhibit IFNγ^+^ γδ T cell responses under these conditions in vitro, supporting IL-2 deprivation as the primary mechanism of suppression (Fig. 3, C and D). By contrast, the proliferation of IL-17^+^ γδ T cells, which was already modest under these stimulation conditions, remained largely unaffected by either Treg cells or IL-2/Neo2/15 supplementation (Fig. 3 E), in agreement with this subset being less dependent on IL-2 signaling (Shibata et al., 2008; Ribot et al., 2012). Furthermore, IL-2 deprivation impaired the cytotoxic function of γδ T cells, as supplementation with IL-2 or Neo2/15 restored their ability to kill E0771 tumor cells, overcoming Treg-mediated suppression in in vitro cytotoxicity assays (Fig. 3 F). These in vitro data clearly suggest that IL-2 deprivation is a key Treg-based mechanism of suppression of anti-tumor type 1 cytotoxic γδ T cells.

### In vivo IL-2 neutralization impairs the therapeutic effect of Treg depletion

**Figure 4.**
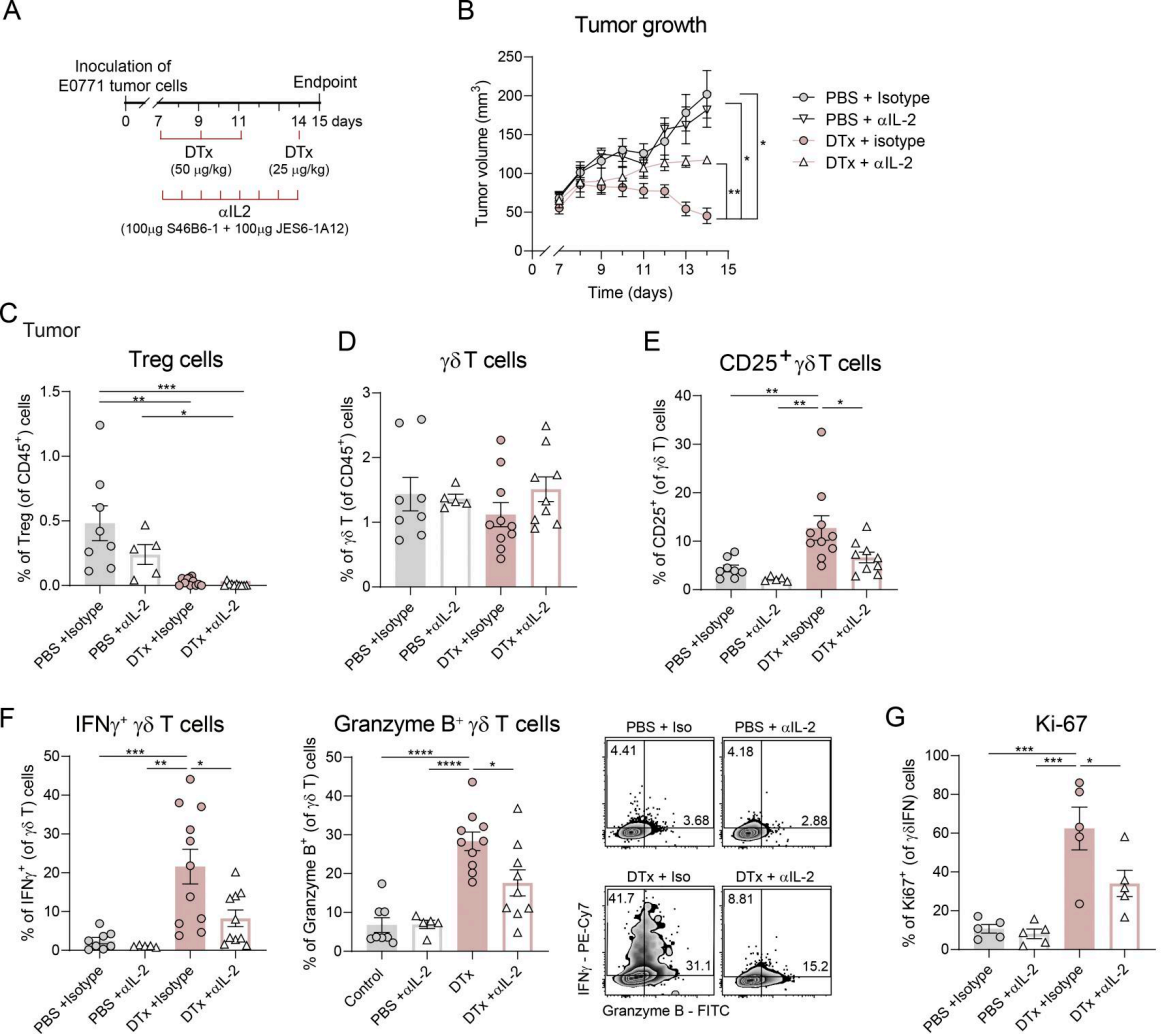
**IL-2 neutralization in the absence of Treg cells limits anti-tumor γδ T cell responses and impairs tumor control. (A)** Schematic representation of the experimental approach. 1 × 10^6^ of E0771 breast cancer cells were inoculated in the mammary fat pad of Foxp3-DTR mice. DTx in PBS was administered intraperitoneally on days 7, 9, 11 (1.5 μg), and 14 (0.75 μg) after tumor inoculation. Between days 7 and 14 a combo of two αIL-2 antibodies (100 μg of S46B6-1 and 100 μg of JES6-1A12) or isotype controls was administered intraperitoneally daily to neutralize IL-2. **(B)** Tumor growth of PBS + isotype- (control), DTx + isotype-, PBS + αIL-2–, or DTx + αIL-2–treated mice (*n* = 5 mice per group). Data are representative of two independent experiments with similar trends and were analyzed by repeated measures two-way ANOVA with Sidak’s multiple comparisons test. **(C)** Percentages of Treg cells in tumors at day 15 after tumor inoculation. **(D and E)** (D) Percentages of γδ T cells within CD45^+^ T cells and (E) CD25 expression within γδ T cells. **(F)** Percentage of IFNγ^+^ cells and granzyme B^+^ cells within γδ T cells and representative density plots. Data in C–F correspond to a pool of two independent experiments with similar trends (*n* = 10 PBS + isotype, 10 DTx + isotype, 5 PBS + αIL-2, or 9 DTx + αIL-2, represented as means ± SEM and analyzed by one-way ANOVA with Tukey’s multiple comparisons test). **(G)** Quantification of proliferation of IFNγ^+^ γδ T cells by Ki-67 expression (*n* = 5 mice per group). One representative out of two independent experiments, analyzed by one-way ANOVA with Tukey’s multiple comparisons test. *P < 0.05, **P < 0.01, ***P < 0.001, and ****P < 0.001.

To determine whether elevated IL-2 availability in the absence of Treg cells drives the in vivo expansion of anti-tumor γδ T cells, we devised a strategy to neutralize IL-2 concomitantly with Treg depletion in E0771 tumor-bearing mice. Beginning on the day of DTx administration, mice were treated daily with equal amounts of two mAbs: one targeting the CD25-binding site of IL-2 (clone S4B6-1) and the other targeting the CD122-binding site (clone JES6-1A12) (Fig. 4 A). This dual blockade ensures effective IL-2 neutralization and prevents the formation of IL-2/anti-IL-2 immune complexes, which paradoxically would enhance receptor signaling by stabilizing the cytokine through its unbound domain (Boyman et al., 2006; McNally et al., 2011; McKinstry et al., 2014). Importantly, this strategy neutralizes IL-2 without directly impacting its receptor. Notably, IL-2 neutralization partially restored tumor growth in Treg-depleted mice, with no changes in Treg-sufficient controls (Fig. 4 B). While the frequencies of tumor-infiltrating Treg cells and total γδ T cells remained minimally affected by transient IL-2 neutralization (Fig. 4, C and D), CD25 upregulation on γδ T cells following Treg-depletion was abrogated upon anti-IL-2 administration, supporting attenuated IL-2 signaling in this population (Fig. 4 E). Accordingly, IL-2 neutralization impaired both αβ T cell responses (Fig. S5 C) and the effector function of γδ T cells, markedly reducing both IFNγ and granzyme B production (Fig. 4 F), as well as the proliferation of IFNγ^+^ γδ T cells (Fig. 4 G). Therefore, enhanced IL-2 availability following Treg depletion unequivocally contributes to the activation and expansion of type 1 cytotoxic γδ T cells within the TME.

### Administration of an IL-2Rβγ_c_ agonist promotes anti-tumor murine γδ T cell responses and tumor control

**Figure 5.**
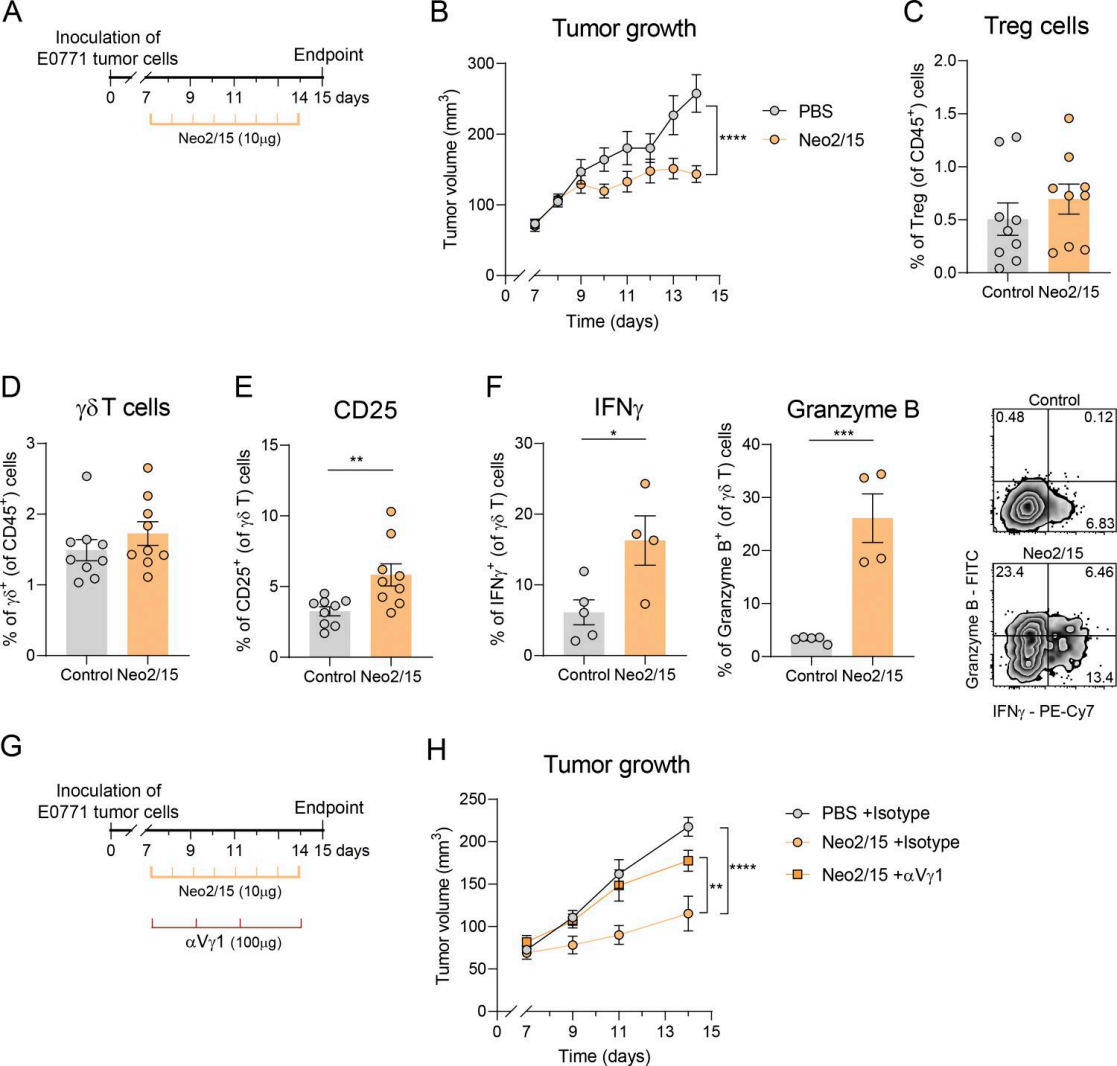
**IL-2Rβ**γ_c_**agonism promotes anti-tumor murine γδ T cell responses and tumor control. (A)** Schematic representation of the experimental approach. 1 × 10^6^ of E0771 breast cancer cells were inoculated in the mammary fat pad of Foxp3-DTR mice (without any DTx administration), and 10 μg of Neo2/15 was administered intraperitoneally daily between days 7 and 14 in the indicated group. **(B)** Tumor growth of Neo2/15-treated or control mice (*n* = 9 mice per group). Data were analyzed by repeated measures two-way ANOVA with Sidak’s multiple comparisons test. **(C)** Percentages of Treg cells in tumors at day 15 after tumor inoculation (*n* = 9 mice per group). **(D and E)** (D) Percentages of γδ T cells within CD45^+^ T cells and (E) CD25 expression within γδ T cells (*n* = 9 mice per group). Data in B–E are represented as means ± SEM of a pool of two independent experiments and analyzed by unpaired *t* test. **(F)** Percentage of IFNγ^+^ cells and granzyme B^+^ cells within γδ T cells and representative density plots (*n* = 5 control mice and 4 Neo2/15-treated mice). Data are represented as means ± SEM of one representative out of two independent experiments and analyzed by unpaired *t* test. **(G)** Schematic representation of the experimental approach to deplete Vγ1^+^ γδ T cells together with Neo2/15 administration. Briefly, 1 × 106 of E0771 breast cancer cells were inoculated in the mammary fat pad of Foxp3-DTR mice. 10 μg of Neo2/15 in PBS was daily administered intraperitoneally. In addition, 100 μg αVγ1 mAb or isotype control was intraperitoneally administered on days 7, 9, 11, and 14 after tumor inoculation. **(H)** Tumor growth of mice treated with isotype (*n* = 5), Neo2/15 (*n* = 5) or Neo2/15 + αVγ1 (*n* = 5), analyzed by repeated measures two-way ANOVA with Sidak’s multiple comparisons test and were representative of two independent experiments. *P < 0.05, **P < 0.01, ***P < 0.001, and ****P < 0.001.

To aim at therapeutic application by overcoming Treg-mediated suppression without going through Treg depletion, and after showing that the IL-2Rβγ_c_ agonist Neo2/15 directly activates type 1 cytotoxic γδ T cells, we treated mice daily with this compound starting on day 7 after tumor inoculation (Fig. 5 A). As expected, Neo2/15 treatment significantly reduced tumor growth (Fig. 5 B). Consistent with previous reports (Silva et al., 2019), Neo2/15 had minimal impact on Treg cell frequency (Fig. 5 C). Although the overall frequency of tumor-infiltrating γδ T cells remained largely unchanged (Fig. 5 D), these cells upregulated CD25 expression following Neo2/15 administration (Fig. 5 E), coinciding with a marked shift toward a type 1 cytotoxic phenotype characterized by increased IFNγ production and granzyme B expression (Fig. 5 F). Importantly, the activation of IFNγ^+^ γδ T cells was substantially responsible for the therapeutic effect of Neo2/15, as depletion of IFNγ-committed Vγ1^+^ γδ T cells significantly impaired Neo2/15-driven tumor control (Fig. 5, G and H). In conclusion, Neo2/15 treatment bypasses Treg-mediated IL-2 sequestration and promotes anti-tumor murine γδ T cell responses contributing to tumor control.

### IL-2Rβγ_c_ agonism circumvents human Treg-mediated suppression and enhances therapeutic γδ T cell responses

**Figure 6.**
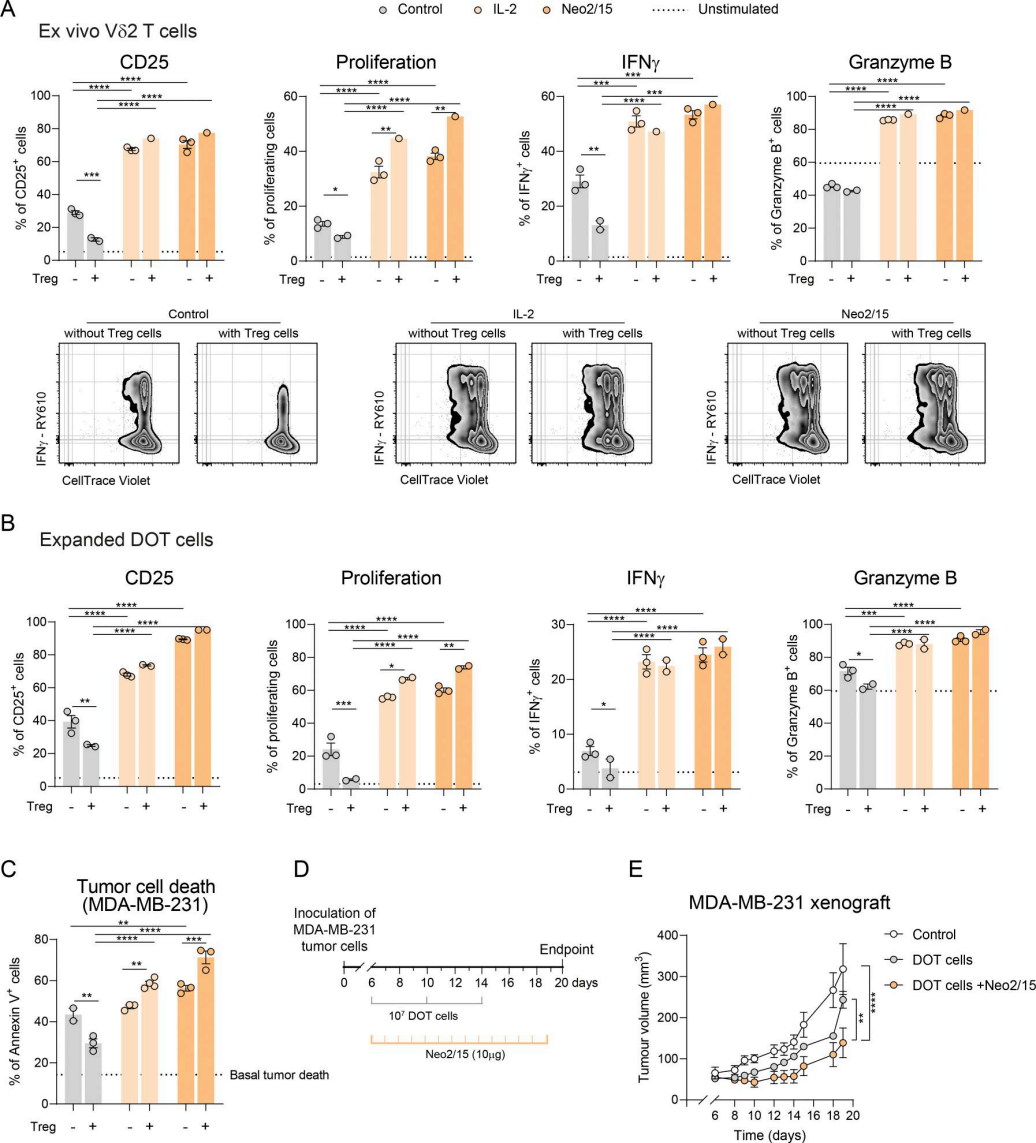
**IL-2Rβγ**_**c**_ **agonism circumvents human Treg suppression and enhances therapeutic γδ T cell responses. (A)** Quantification and representative density plots of proliferating sorted Vδ2^+^ T cells (measured by dilution of CellTrace Violet staining), as well as their CD25, IFNγ, and granzyme B expression after 3 days in culture with plate bound anti-CD3 and soluble anti-CD28 mAbs, with or without sorted Treg cells from human PBMCs and in the presence or absence of IL-2 or Neo2/15. Dotted line represents baseline levels of unstimulated cells (no anti-CD3/28). **(B)** Quantification of proliferation and expression of IFNγ and granzyme B in DOT cells under the above-mentioned conditions. **(C)** Representation of tumor cell death, quantified by annexin V staining after 3-h killing assay following preincubation in the above-mentioned conditions. Dotted line represents baseline tumor death. Data in A–C represented as means ± SEM, are representative of three independent experiments with different donors and analyzed by two-way ANOVA with Sidak’s multiple comparisons test. **(D)** Schematic representation of the orthotopic xenograft breast cancer tumor model. 1 × 10^6^ of MDA-MB-231 cells were implanted subcutaneously in the mammary fat pad. After confirmation of tumor engraftment on day 6, mice were treated with three intravenous injections of 5–10 × 10^6^ DOT cells 4 days apart. Some mice were treated with 10 µg of Neo2/15 daily. **(E)** Tumor growth over time (*n* = 5 mice per group). Data represent means ± SEM, are representative of two independent experiments and were analyzed by repeated-measures two-way ANOVA with Sidak’s post hoc test. Only statistical differences at day 18 are represented by asterisks. *P < 0.05, **P < 0.01, ***P < 0.001, and ****P < 0.001.

To determine whether the Treg suppression mechanism found in mice is conserved in humans, we isolated Treg cells (sorted as CD4^+^CD25^+^CD127^−^) and γδ T cells (sorted as CD4^−^TCRVδ1^−^TCRVδ2^+^, corresponding to the Vδ2 subset that predominates in circulation) from human peripheral blood mononuclear cells (PBMCs) and co-cultured them in the presence or absence of recombinant human IL-2 or Neo2/15. Consistent with our findings with mouse cells, human Treg cells prevented CD25 upregulation and inhibited both the proliferation and IFNγ production of Vδ2^+^ γδ T cells (Fig. 6 A and Fig. S5 D). Notably, this suppression was abolished by adding exogenous IL-2 or Neo2/15, which were both able to stimulate potent Vδ2^+^ γδ T cell proliferation and effector functions even in the presence of Treg cells (Fig. 6 A and Fig. S5 D).

Finally, we hypothesized that Treg cells could represent a hurdle to emerging adoptive γδ T cell therapies. Our laboratory previously developed Delta One T (DOT) cells, a Vδ1-enriched γδ T cell therapy product generated upon expansion of blood-derived human γδ T cells, and demonstrated its efficacy in xenograft models of leukemias and colorectal cancer (Almeida et al., 2016; Di Lorenzo et al., 2019; Blanco-Domínguez et al., 2025; Mensurado et al., 2024). We now found that Treg cells are able to suppress the activation and proliferation of DOT cells in vitro and that supplementation with IL-2 or Neo2/15 fully restored their activity (Fig. 6 B). Moreover, the cytotoxic function of DOT cells against MDA-MB-231 triple-negative breast cancer cells was impaired following co-culture with Treg cells, while stimulation of the IL-2 receptor via Neo2/15 bypassed Treg-mediated suppression (Fig. 6 C).

To assess whether IL-2Rβγ_c_ engagement could enhance the therapeutic activity of DOT cells in vivo, we orthotopically implanted MDA-MB-231 breast cancer cells into the mammary gland of NSG mice engineered to produce basal levels of human IL-15. Mice were treated intravenously with DOT cells, with or without concomitant intraperitoneal administration of Neo2/15 (Fig. 6 D). While DOT cells alone achieved partial tumor control, co-administration of Neo2/15 significantly enhanced their anti-tumor efficacy (Fig. 6 E).

Collectively, these findings indicate that human Treg cells can suppress the activity of both endogenous (Vδ2) and expanded (DOT) γδ T cells and that promoting IL-2Rβ signaling can boost γδ T cell/DOT cell performance in adoptive cell therapy.

## Discussion

γδ T cells are increasingly recognized as important effectors in anti-tumor immunity, particularly through their capacity to produce abundant IFNγ and exert potent cytotoxicity in complementary modes to αβ T cells or NK cells (Hayday et al., 2024). In this study, we unraveled a novel immunoregulatory axis between Treg cells and anti-tumor γδ T cells, which can be targeted via IL-2Rβγ_c_ signaling to enhance the efficacy of γδ T cell–based cancer immunotherapy.

First focusing on a syngeneic mouse model of breast cancer, we found that induced Treg depletion unleashed the proliferation of IFNγ-committed (mostly Vγ1^+^) cells in the tumor bed. Importantly, Treg cell depletion did not impact γδ T cell subsets biased toward IL-17 production. We associated this differential impact on γδ T cell subsets to the corresponding expression of distinct IL-2R chains, which determine binding affinity, and functional dependence on IL-2 signaling. Nevertheless, we acknowledge that the E0771 and MC38 models used in the study are not particularly well-suited to study IL-17^+^ γδ T cells, since they are biased toward an IFN-γ response derived from activated Vγ1^+^ cells. In addition, a limitation of the E0771 breast cancer model is that it does not allow discrimination between γδ T cell responses originating from the mammary gland epithelium versus those arising from the surrounding fat pad, thus the precise tissue source of the proliferating IFNγ^+^ γδ T cells was not determined.

We demonstrate that Treg cells suppress tumor-associated IFNγ^+^ γδ T cells by limiting their access to IL-2 that is critical for their proliferation and effector functions. Although Treg cells cannot produce IL-2 themselves, they depend on it for their survival and function, and their constitutive expression of the high-affinity IL-2 receptor incorporating the α chain (CD25) allows them to efficiently sequester this cytokine, thereby limiting its availability to effector T cells and suppressing their activation (Pandiyan et al., 2007). A genetic approach to uncouple IL-2 signaling from Treg function by expressing a constitutively active STAT5b in IL-2R–deficient Treg cells revealed that IL-2 limitation is necessary to restrain CD8^+^ T cell expansion (Chinen et al., 2016). In CD4^+^ T cells, this mechanism limits IFNγ and granzyme B expression via T-bet and Blimp-1, respectively (Śledzińska et al., 2020). On the other hand, CD8^+^ T cell expansion was promoted by a mAb specific for IL-2 administered in combination with recombinant IL-2, leading to an increased biological activity via the formation of stabilizing complexes (Boyman et al., 2006). Moreover, Treg depletion with a CD25 antibody that preserves IL-2 bioactivity led to augmented CD8^+^ T cell responses and enhanced anti–PD-1 mAb therapeutic activity (Galvez-Cancino et al., 2025).

Our data now extend this “IL-2-sink” mechanism to γδ T cells, positioning IL-2 competition as a key regulatory mechanism against IFNγ^+^ γδ T cells. Previous work demonstrated that autocrine IL-2 production is essential for mouse γδ T cell survival and proliferation (Ribot et al., 2012) and that stimulation with IL-2–induced IFNγ and cytolytic activity in human γδ thymocytes (Ribot et al., 2014). In line with this, we now show that increased IL-2 availability promotes robust expansion of IFNγ^+^ γδ T cells, reinforcing the notion that IL-2 is a critical limiting factor for their activation and anti-tumor function. We note, however, that Treg depletion also induces strong αβ T cell activation. In this regard, concomitant depletion of Treg cells and CD8^+^ T cells did not impair the accumulation of IFNγ^+^ γδ T cells, indicating that CD8^+^ T cells are dispensable for this response, although a contribution from CD4^+^ T cells, as a major source of IL-2, remains probable. Interestingly, the depletion of the main IFNγ^+^ γδ T cell subset, Vγ1^+^ cells, resulted in reduced frequencies of IFNγ-producing CD4^+^ and CD8^+^ αβ T cells, consistent with our previous findings in a malaria infection model (Ribot et al., 2019) and with the established concept that γδ T cells modulate broader immune responses (Vantourout and Hayday, 2013; Arias-Badia et al., 2024).

Recent advances in cytokine engineering offer promising tools to circumvent Treg-mediated suppression. For example, an IL-2 variant (AB248) selectively targeted to CD8^+^ T cells was recently developed, enabling robust effector T cell responses with limited Treg or NK cell activation (Moynihan et al., 2024). Another example of cytokine engineering is the development of the Neo 2/15 agonist used in this study, which takes advantage of the differential signaling properties of IL-2 and IL-15. While IL-2 signals through the trimeric high-affinity receptor (IL-2Rαβγ_c_), primarily expressed by Treg cells and activated T cells, IL-15 signals through the shared IL-2Rβγ_c_ complex and is presented in trans by IL-15Rα–expressing cells (Tamzalit et al., 2014). Our data with Neo2/15, which binds to the IL-2Rβγ_c_ heterodimer and elicits downstream cell signaling independent of IL-2Rα and IL-15Rα, clearly showed its ability to circumvent Treg-mediated inhibition of both mouse and human γδ T cells, thus providing the proof of concept for its use in combination with upcoming γδ T cell–based therapies, including DOT cells. These are expanded in response to IL-2 or IL-15, and the latter is essential for their enhanced cytotoxic capacity (Almeida et al., 2016) and for in vivo persistence (Sánchez Martínez et al., 2022). Although other lymphocyte subsets may also respond to Neo2/15, our in vitro and in vivo data firmly establish mouse and human γδ T cells as direct targets of this engineered cytokine. Thus, the co-administration of Neo2/15 with adoptive DOT cell therapy may enhance clinical efficacy, in line with our preclinical data obtained using the MDA-MB-231 triple-negative breast cancer xenograft model. We acknowledge, however, that this xenograft model lacks Treg cells, whose modulatory effects on γδ T cell responses were therefore only assessed in vitro, representing a limitation of our study. Interestingly, CAR-NK cells engineered to secrete Neo2/15 enhanced their cytotoxicity and persistence in different solid tumor models, suggesting that similar armoring strategies on DOT (or other γδ T) cell therapies may also bypass TME immunosuppression (Luo et al., 2025).

Beyond IL-2 competition, Treg cells may suppress γδ T cells through additional pathways, as suggested by studies in other murine and human disease contexts. Treg-derived mediators such as CTLA-4, IL-10, IL-35, and adenosine have been shown to selectively suppress IL-17^+^ γδ T cells in models of psoriasis (Lee et al., 2023; Sivasami et al., 2023), colitis (Park et al., 2010; Yurchenko et al., 2011), lung inflammation (Faustino et al., 2020; Xu et al., 2023), and myocardial infarction (Blanco-Domínguez et al., 2022), while Glucocorticoid-Induced TNFR-Related protein (GITR) was shown to impact both IL-17^+^ and IFNγ^+^ γδ T cells in the context of malaria infection (Gonçalves-Sousa et al., 2010). With regard to human γδ T cells, they were inhibited in vitro by Treg-derived IL-10 and TGF-β in tuberculosis and hepatocellular carcinoma (Yi et al., 2013; Li and Wu, 2008). Our in vivo data confirm an overtly dominant role of IL-2 deprivation specifically preventing anti-tumor γδ T cell responses in our study, although these alternative mechanisms might also play a role in other cancer settings.

Collectively, our findings identify a previously underappreciated axis of regulation in which Treg cells constrain anti-tumor γδ T cells through IL-2 cytokine competition. This adds to the growing body of evidence that the suppressive capacity of Treg cells extends beyond conventional T cells and positions IL-2 bioavailability as a key modulator of γδ T cell function in cancer. Therapeutic strategies that deplete or functionally disable Treg cells, or that selectively deliver cytokine signaling to effector cells, such as the Neo2/15 agonist explored here, may thus be the key to unlocking the full potential of γδ T cells in cancer immunotherapy.

## Materials and methods

### Mice

C57BL/6J Foxp3-DTR (B6.129 [Cg]-Foxp3tm3 [DTR/GFP] Ayr/J) (Foxp3-DTR [C57BL/6 background] [Kim et al., 2007]) were obtained from The Jackson Laboratory and are backcrossed for at least eight generations to C57BL/6NTac mice, and NOD.Cg-Prkdcscid Il2rgtm1Wjl Tg(IL15)1Sz/SzJ (NSG-Tg[Hu-IL15] [NOD/ShiLtJ background]) were obtained from The Jackson Laboratory, and C57BL/6J FOXP3-hCD2/IL-17A-GFP (CD57BL/6 background) mice were bred in house from C57BL/6J FOXP3-hCD2 mice, kindly provided by Prof. Shohei Hori (University of Tokyo, Japan), and C57BL/6J IL-17A-GFP mice were obtained from Biocytogen. Mice between 6 and 20 wk old were used in all experiments. All the in vivo experiments were performed in female mice due to the biological relevance of breast cancer. Control mice used throughout the study were of the same strain (C57BL/6J Foxp3-DTR) but were not injected with DTx. Both male and female mice were used for ex vivo co-culture experiments. Mice were maintained under a 14-h/10-h light/dark cycle, with access to food and water ad libitum and controlled temperature and humidity conditions. All mice were kept at Gulbenkian Institute for Molecular Medicine (GIMM)’s rodent facility in a specific pathogen–free environment.

All experiments with mice were approved by the Animal Welfare Body of the institute (Órgão Responsável pelo Bem-Estar Animal (ORBEA)-GIMM) and by the national competent authority, Direção Geral de Alimentação e Veterinária, according to national and European regulations.

### Syngeneic orthotopic breast cancer model

E0771 breast cancer cell line was purchased from Tebubio. Cells were maintained in DMEM (Life Technologies) with glutamine, sodium pyruvate, 10% FCS (Life Technologies), and 1% penicillin-streptomycin at 37°C and 5% CO_2_. For inducing orthotopic breast tumors, either C57BL/6J Foxp3-DTR or wild-type mice were injected with 1 × 10^6^ viable E0771 cells in 100 μl of 1:1 PBS:Matrigel solution (Corning Matrigel Matrix) in the fat pad of the fourth left nipple, directly under the nipple. One week after tumor implantation, tumors were measured using a digital caliper, and mice were equally distributed in experimental groups. To deplete Treg cells, C57BL/6J Foxp3-DTR mice received intraperitoneal injections of 1.5 μg DTx in 100 μl of PBS at days 7, 9, and 11 after tumor inoculation, followed by a last injection of 0.75 μg at day 13/14. Animals were monitored daily or every 2 days from day 7, and their weight and tumor volume were assessed to guarantee the well-being of the animal. Tumor volumes were estimated following the formula width^2^ × length/2 (Faustino-Rocha et al., 2013). Mice were sacrificed on day 15 after the tumor implantation. When indicated, either Vγ1^*+*^ T cells or CD8 T cells were depleted by intraperitoneal injection of either 100 µg αVγ1.1/Cr4 (clone 2.11; BioXcell) or αCD8β (clone Ly3.2; BioXcell) mAbs, respectively, in 100 μl PBS on days 7, 9, 11, and 14. In vivo IL-10 neutralization was induced with the injection of 200 µg of anti-IL-10 mAb (clone JES5-2A5; BioXcell) (or isotype control) intraperitoneally at days −1, 0, 1, and 3 after tumor inoculation and intra-tumoral at days 7, 10, and 13. In vivo IL-2 neutralization was induced by daily intraperitoneal injections of anti-IL-2 mAbs (S6-1A12 and S4B6 clones, 100 µg each, both from BioXcell) in 100 μl PBS. IL-2Rβ agonism was achieved by daily intraperitoneal injections of 10 µg Neo2/15 (Silva et al., 2019) in 50 μl PBS. For in vivo proliferation assays, the animals were injected intraperitoneally with 1.5 mg of BrdU (Sigma-Aldrich) diluted in PBS on days 7, 9, 11, and 13. Additionally, BrdU was also incorporated in the drinking water of these animals (0.8 mg/ml) since day 7 of tumor injection.

### Syngeneic colorectal cancer model

MC38 cell line was purchased from Kerafast. Cells were maintained in DMEM with glutamine, nonessential amino acids, 10% FCS (Life Technologies), and 1% penicillin-streptomycin at 37°C and 5% CO_2_. For tumor inoculation, C57BL/6J Foxp3-DTR female mice were injected with 2 × 10*6* viable MC38 cells in 100 μl of 1:1 PBS:Matrigel solution (Corning Matrigel Matrix) in the right flank subcutaneously. Six days after tumor implantation, tumors were measured using a digital caliper, and mice were equally distributed in experimental groups. To deplete Treg cells, C57BL/6J Foxp3-DTR mice received intraperitoneal injections of 1.5 μg DTx in 100 μl of PBS at days 6, 8, and 10 after tumor inoculation, followed by a last injection of 0.75 μg at day 12. Animals were monitored daily or every 2 days from day 7, and their weight and tumor volume were assessed to guarantee the well-being of the animal. Tumor volumes were estimated following the formula width ^2^ × length/2 (Faustino-Rocha et al., 2013). Mice were sacrificed on day 13 after the tumor implantation.

### Xenograft orthotopic breast cancer model

MDA-MB-231 cancer cell line was purchased from ATCC. Cells were maintained in DMEM (Life Technologies) with glutamine, sodium pyruvate, 10% FCS (Life Technologies), and 1% penicillin-streptomycin at 37°C and 5% CO_2_. For inducing orthotopic breast tumors, NOD.Cg-Prkdcscid Il2rgtm1Wjl Tg(IL15)1Sz/SzJ (Jackson Laboratories) were injected with 1 × 10^6^ viable MDA-MB-213 cells in 100 μl of 1:1 PBS:Matrigel solution (Corning Matrigel Matrix) in the fat pad of the fourth left nipple, directly under the nipple. One week after tumor implantation, tumors were measured using a digital caliper, and mice were equally distributed in experimental groups. Mice received 5–10 × 10^6^ viable DOT cells intravenously at the time points indicated in the figure and daily intraperitoneal injections of 10 µg Neo2/15 (Silva et al., 2019) in 50 μl PBS. Tumor volumes were estimated following the formula width^2^ × length/2

### Flow cytometry analysis

For analysis of tumor infiltrates, the tumor was harvested and cut into small pieces. 100 μg/ml of DNAse I (Roche), 1 mg/ml of collagenase (I) (Roche), and 0.4 mg/ml of collagenase (IV) were used to digest the tumor for 30 min at 37°C. After that time, the digested sample was filtered through a 100-μm cell strainer. For the analysis of the spleen and dLN, the organs were mashed and filtered through a 40-μm cell strainer. Cells were counted and transferred to a V-bottom 96-well plate where they were stimulated at a maximum density of 4 × 10^6^ cells/well in 200 μl of complete RPMI medium with PMA (50 ng/ml, Ref: 16561-29-8; Sigma-Aldrich) and ionomycin (1 μg/ml, Ref: 56092-81-0; Sigma-Aldrich) in the presence of Brefeldin A (BFA; 10 μg/ml, Ref: 20350-15-6; Sigma-Aldrich) and Monensin (Invitrogen eBioscience Monensin Solution [1000X] Ref: 00-4505-51) for 3–4 h at 37°C, 5% CO_2_. The cells were then washed in FACS buffer (79.6% H_2_O; 10% PBS 1 M; 10% heat-inactivated FCS; 0.4% ultrapure EDTA). For surface staining, cells were incubated with anti-mouse CD16/CD32 (1:200, Ref: 14-0161-82; Thermo Fisher Scientific) concomitantly with the surface marker staining (antibody clones in Table 1) that was performed by adding the appropriate antibody mix in FACS buffer (20 min, 4°C). LIVE/DEAD Fixable Near-IR Dead Cell Stain (Invitrogen Ref: L10119) dye was also added in this step. For intracellular cytokine staining, cells were then fixed and permeabilized using eBioscience Foxp3/Transcription Factor Fixation/Permeabilization, following the manufacturer’s instructions, and then incubated for 1 h with mAbs to detect cytokine production. Antibodies were purchased from eBioscience, BioLegend, and Invitrogen as summarized in Table 1. For BrdU staining, fluorescein isothiocyanate BrdU Flow Kit (Ref: 559619; BD Pharmingen) was used following the manufacturer’s instructions. Flow cytometry data acquisition was performed using the BD LSRFortessa X-20 (2010; BD Biosciences) or FACSymphony A5 SE (2023; BD Biosciences) cell analyzers and the FACSDiva software (version 6.2, BD Biosciences). Data were analyzed using the FlowJo software (version 10.8.1, BD Biosciences). Clustering of γδ T cells based on spectral flow cytometry data was performed with the FlowSOM plugin for FlowJo (version 4.1.0) considering the expression following markers: Vγ1, Vγ4, CD25, CD122, CD4, CD8, T-bet, RORγt, granzyme B, IFNγ, IL-17A, and Ki-67.

**Table 1.** List of antibodies and dyes used for flow cytometry analyses

Target species	Marker	Fluorochrome	Dilution factor	Clone	Manufacturer	Catalog number
Human	CD127	PE	100	HIL-7R-M21	BD Biosciences	557938
Human	CD2	APC	100	RPA-2.10	Invitrogen	17-0029-42
Human	CD3	BUV805	200	SK7	BD Biosciences	612894
Human	CD4	APC	200	RPA-T4	eBioscience	17-0049--42
Human	CD4	APCCy7	200	RPA-T4	BioLegend	300518
Human	GzmB	RY703	200	GB11	BD Biosciences	571462
Human	IFNg	RY610	200	B27	BD Biosciences	571143
Human	Perf	BV711	100	DG9	Sony	2140645
Human	TNFa	BUV395	200	MAb11	BD Biosciences	563996
Human	Vd1	FITC	200	REA173	Miltenyi	130-118-362
Human	Vd2	PercpCy5.5	200	B6	BioLegend	331423
Mouse	CD11b	Percp5.5	200	M1/70	BioLegend	101228
Mouse	CD122	FITC	100	TM-β1	BioLegend	123208
Mouse	CD122	RB780	200	TM-β1	BD Biosciences	755581
Mouse	CD25	BV605	200	PC61	BioLegend	102035
Mouse	CD25	APC-eF780	50	*PC61.5*	Invitrogen	47-0251-82
Mouse	CD3	PECy7	50	145-2C11	BioLegend	100320
Mouse	CD3	BV711	100	17A2	BioLegend	100241
Mouse	CD3	RB744	200	145-2C11	BD Biosciences	757837
Mouse	CD4	RB613	200	GK1.5	BD Biosciences	571102
Mouse	CD45	RB705	400	30-F11	BD Biosciences	570290
Mouse	CD8a	BV510	200	53–6.7	BioLegend	100752
Mouse	Foxp3	AF700	100	FJK-16 s	Invitrogen	56-5773-80
Mouse	Granzyme B	FITC	200	GB11	BioLegend	515403
Mouse	IFNg	PEDzzle	200	XMG1.2	BioLegend	505845
Mouse	IFNg	PECy7	100	XMG1.2	Invitrogen	45-7311-82
Mouse	IL-17	BUV395	100	TC11-18H10	BD Biosciences	565246
Mouse	Ki67	BV605	250	16A8	BioLegend	652413
Mouse	Rorgt	AF647	100	Q31-378	BD Biosciences	562682
Mouse	Tbet	BV711	100	4B10	BioLegend	644820
Mouse	TCRgd	SB780	500	eBioGL3	Invitrogen	78-5711-82
Mouse	TCRgd	BV421	100	GL3	BioLegend	118120
Mouse	Vg1	BV650	100	2.11	BD Biosciences	745310
Mouse	Vg4	PercpCy5.5	200	UC3-10A6	BioLegend	137712
Mouse	Vg4	FITC	100	UC3-10A6	BioLegend	137704
Mouse	Vg4	PE	100	UC3-10A6	BioLegend	137706
Mouse	Stat5(pY694)	PE	20	47/Stat5(pY694)	BD Biosciences	562077
​	Viability	Zombie Yellow	500	​	BioLegend	423103
​	Viability	Zombie Aqua	500	​	BioLegend	423101

### In vitro co-cultures of γδ T cells and Treg cells from mice

Spleen and LNs (superficial cervical, brachial, axillary, and inguinal) were harvested from reporter Foxp3-hCD2/IL-17A-GFP mice and mashed onto 40-μm cell strainers to generate cell suspensions. RBCs were lysed using RBC Lysis Buffer (BioLegend) and then incubated with anti-TER119, anti-CD11b, and anti-CD19 for 20 min at 4 °C. Subsequently, cells were incubated with anti-biotin microbeads (Miltenyi Biotec) and negatively enriched using LS columns (Miltenyi Biotec) following the manufacturer’s instructions. Negative fractions were stained with viability dye and fluorescence-conjugated antibodies against mouse TCRγδ (clone eBioGL3; eBioscience), mouse CD3e (clone 145-2C11; eBioscience), and human CD2 (clone RPA-2.10; Invitrogen) for 20 additional minutes on ice. Then, γδ T cells were sorted as alive CD3^+^TCRγδ^+^, whereas Treg cells were sorted as alive CD3^+^hCD2^+^ cells. After sorting, γδ T cells were stained with CellTrace Violet (Thermo Fisher Scientific) at a concentration of 2.5 µM in PBS for 15 min in the dark at room temperature, after which cells were washed and cultured. γδ T cells and Treg cells were cultured for 3 days in a 96-well round-bottom plate coated with anti-CD3 (2.5 μg/ml, clone 145-2C11; BioLegend) together or separated in complete RPMI with IL-7 (20 ng/ml, PeproTech), anti-CD28 (2.5 μg/ml, clone 37.51; Invitrogen), and in the presence or absence of recombinant murine IL-2 (10 ng/ml, PeproTech) or Neo2/15 (10 ng/ml [Silva et al., 2019]). Cells were cultured for 72 and 3 h before they were incubated with PMA (50 ng/ml, Sigma-Aldrich) and ionomycin (1 μg/ml, Sigma-Aldrich) in the presence of BFA (10 μg/ml, Sigma-Aldrich) and Monensin (Invitrogen eBioscience Monensin Solution [1000X]). Following incubation, cells were washed and analyzed by flow cytometry. For all culturing steps, the complete RPMI refers to RPMI (Gibco) supplemented with 10% FCS, 1% penicillin-streptomycin, 10% HEPES, 10% Sodium Pyruvate, 10% non-essential amino acids solution, 0.1% Gentamycin, and 0.1% 2-Mercaptoethanol (all from Gibco). For phosphorylated STAT-5 stainings, sorted γδ T cells and Treg cells were re-stained with surface markers and cultured together or separated for 10 min at 37° and 5% CO_2_ with/without 10 ng/ml recombinant IL-2 (PeproTech) or Neo2/15 (Silva et al., 2019). Then, the medium was removed, and cells were fixed for 30 min and intranuclearly stained with PE-conjugated anti-STAT5 pY694 (clone 47/Stat5(pY694); BD Biosciences) for 30 additional minutes using the BD Phosflow protocol (BD Biosciences).

### In vitro co-cultures of γδ T cells and Treg cells from humans

Buffy coats from healthy volunteers were obtained under the agreement (15.12.2003) between GIMM and Instituto Português do Sangue e da Transplantação. These procedures are authorized by the respective authorities, namely the Autoridade para os Serviços de Sangue e Transplantação and the Comissão Nacional de Proteção de Dados, and follow the applicable national and European regulations, including EU Directives 2004/33/EC, 2005/61/EC, and 2005/62/EC, and the General Data Protection Regulation (EU) 2016/679. All experimental work involving human cells complies with all ethical principles and with European Directive 2004/23/EC and Portuguese Law No. 12/2009, which establish standards for the quality and safety of human cells used in research.

Human PBMCs from healthy donors were stained for 20 min on ice with viability dye and fluorochrome-conjugated antibodies against CD4, TCRVδ1 (REA173, Miltenyi), TCRVδ2, CD25, and CD127 (clones and references listed in Table 1). Treg cells were sorted as alive CD4^+^CD25^+^CD127^−^ cells, whereas Vδ2 cells were sorted as CD4^−^TCRVδ1^−^TCRVδ2^+^ cells from the same PBMC sample.

In addition, we used DOT cells expanded as previously described. In brief, αβ T cell–depleted were seeded in G-REX vessels (Wilson Wolf Manufacturing). The culture medium used was OpTmizer-CTS, enriched with 2.5% heat-inactivated human plasma (LifeSciences), 2 mM L-glutamine (Thermo Fisher Scientific), and penicillin/streptomycin at concentrations of 50 U/ml and 50 µg/ml, respectively (Thermo Fisher Scientific). On day 0, the following recombinant, animal-free human cytokines were added to the culture: IL-4 (100 ng/ml), IFNγ (70 ng/ml), IL-21 (7 ng/ml), IL-1β (15 ng/ml), and a soluble anti-CD3 mAb (140 ng/ml, clone OKT-3; BioLegend), all sourced from PeproTech unless otherwise noted. On day 7, cultures were further supplemented with additional anti-CD3 (clone OKT-3, 1 µg/ml), IL-21 (13 ng/ml), and IL-15 (70 ng/ml). On day 11, cultures received fresh medium containing anti-CD3 (1 µg/ml) and IL-15 (100 ng/ml). Cells were maintained for 14 days at 37°C in a 5% CO_2_ atmosphere. DOT cells were collected at the end of the culture period and used either fresh for in vitro applications or cryopreserved for in vivo use. For cryopreservation, cells were resuspended in CryoStor cell cryopreservation media (Merck) and then stored in liquid nitrogen.

Then, sorted Vδ2 cells and expanded DOT cells were cultured alone or in the presence of sorted Treg cells for 3–4 days in a 96-well round-bottom plate coated with anti-CD3 (2.5 μg/ml, clone OKT3; BioLegend) in complete RPMI with anti-CD28 (2.5 μg/ml, clone CD28.2; Invitrogen) in the presence or absence of recombinant human IL-2 (PeproTech) or Neo2/15 (Silva et al., 2019) at 10 ng/ml. Following co-culture, Treg cells were depleted by staining with APC-conjugated anti-CD4 (clone RPA-T4; eBioscience), followed by magnetic separation using anti-APC microbeads and MS columns (Miltenyi). The remaining DOT cells were subsequently co-cultured with CellTrace Violet–labeled MDA-MB-231 breast cancer cells in complete RPMI for 3 h at a 5:1 effector-to-target ratio. Tumor cell death was assessed by annexin V staining (Alexa Fluor 647; BioLegend).

### Statistical analysis

For statistical analysis, the normality of data distributions was evaluated using the Shapiro–Wilk test. When data followed a normal distribution, two-group comparisons were performed with an unpaired Student’s *t* test (or a paired *t* test for matched samples), while comparisons across >2 groups were assessed using one-way ANOVA followed by Tukey’s multiple comparisons test. For non-normally distributed data, two-group comparisons were conducted using the Mann–Whitney U test, and multiple-group comparisons were analyzed with the Kruskal–Wallis test followed by Dunn’s multiple comparisons test. In kinetic experiments or when analyzing two pairs of groups, a two-way ANOVA with Šidák’s post hoc multiple comparisons test was applied. Data analysis was performed using GraphPad Prism 9 software (GraphPad Software Inc.). The applied tests are indicated in the figure legends, where data are expressed as mean ± standard deviation, and P < 0.05 was considered significant. Results are presented as P values * = P < 0.05, ** = P < 0.01, *** = P < 0.001, and **** = P < 0.0001.

### Online supplemental material

Fig. S1 is related to Fig. 1 and contains additional data on IFNγ^+^ γδ T cell expansion upon Treg cell depletion in the MC38 colon cancer model, as well as in tumor-free mice, including proliferation (Ki-67) and tissue distribution analyses. Fig. S2 is related to Figs. 1 and 2 and shows the systemic increase of IFNγ-producing αβ T cells following Treg depletion, as well as functional data supporting the contribution of CD8 T cells to tumor control. Fig. S3 is related to Fig. 3 and includes experiments assessing alternative suppressive pathways, demonstrating that blockade of IL-10, IL-35, or adenosine signaling does not impair Treg-mediated suppression of IFNγ^+^ γδ T cells. Fig. S4 is related to Fig. 3 and provides detailed characterization of IL-2 receptor subunit expression (CD25 and CD122) across γδ T cell subsets and Treg cells in steady-state and tumor-bearing conditions. Fig. S5 is related to Figs. 3, 4, and 5 and contains additional data on IL-2 signaling, including STAT5 phosphorylation, the impact of IL-2 neutralization on αβ T cell responses and presents donor-specific data demonstrating the suppressive effect of human Treg cells on γδ T cell proliferation and effector function across independent donors.

## Data Availability

Data supporting the findings of this study are available from the corresponding authors on reasonable request.
